# Gluconeogenesis is essential for trypanosome development in the tsetse fly vector

**DOI:** 10.1371/journal.ppat.1007502

**Published:** 2018-12-17

**Authors:** Marion Wargnies, Eloïse Bertiaux, Edern Cahoreau, Nicole Ziebart, Aline Crouzols, Pauline Morand, Marc Biran, Stefan Allmann, Jane Hubert, Oriana Villafraz, Yoann Millerioux, Nicolas Plazolles, Corinne Asencio, Loïc Rivière, Brice Rotureau, Michael Boshart, Jean-Charles Portais, Frédéric Bringaud

**Affiliations:** 1 Laboratoire de Microbiologie Fondamentale et Pathogénicité (MFP), Université de Bordeaux, CNRS UMR-5234, Bordeaux, France; 2 Centre de Résonance Magnétique des Systèmes Biologiques (RMSB), Université de Bordeaux, CNRS UMR-5536, Bordeaux, France; 3 Trypanosome Transmission Group, Trypanosome Cell Biology Unit, Department of Parasites and Insect Vectors and INSERM U1201, Institut Pasteur, Paris, France; 4 LISBP, Université de Toulouse, CNRS, INRA, INSA, Toulouse, France; 5 Fakultät für Biologie, Genetik, Ludwig-Maximilians-Universität München, Martinsried, Germany; Oregon Health & Science University, UNITED STATES

## Abstract

In the glucose-free environment that is the midgut of the tsetse fly vector, the procyclic form of *Trypanosoma brucei* primarily uses proline to feed its central carbon and energy metabolism. In these conditions, the parasite needs to produce glucose 6-phosphate (G6P) through gluconeogenesis from metabolism of non-glycolytic carbon source(s). We showed here that two phosphoenolpyruvate-producing enzymes, PEP carboxykinase (PEPCK) and pyruvate phosphate dikinase (PPDK) have a redundant function for the essential gluconeogenesis from proline. Indeed, incorporation of ^13^C-enriched proline into G6P was abolished in the PEPCK/PPDK null double mutant (Δ*ppdk*/Δ*pepck*), but not in the single Δ*ppdk* and Δ*pepck* mutant cell lines. The procyclic trypanosome also uses the glycerol conversion pathway to feed gluconeogenesis, since the death of the Δ*ppdk*/Δ*pepck* double null mutant in glucose-free conditions is only observed after RNAi-mediated down-regulation of the expression of the glycerol kinase, the first enzyme of the glycerol conversion pathways. Deletion of the gene encoding fructose-1,6-bisphosphatase (Δ*fbpase*), a key gluconeogenic enzyme irreversibly producing fructose 6-phosphate from fructose 1,6-bisphosphate, considerably reduced, but not abolished, incorporation of ^13^C-enriched proline into G6P. In addition, the Δ*fbpase* cell line is viable in glucose-free conditions, suggesting that an alternative pathway can be used for G6P production *in vitro*. However, FBPase is essential *in vivo*, as shown by the incapacity of the Δ*fbpase* null mutant to colonise the fly vector salivary glands, while the parental phenotype is restored in the Δ*fbpase* rescued cell line re-expressing FBPase. The essential role of FBPase for the development of *T*. *brucei* in the tsetse was confirmed by taking advantage of an *in vitro* differentiation assay based on the RNA-binding protein 6 over-expression, in which the procyclic forms differentiate into epimastigote forms but not into mammalian-infective metacyclic parasites. In total, morphology, immunofluorescence and cytometry analyses showed that the differentiation of the epimastigote stages into the metacyclic forms is abolished in the Δ*fbpase* mutant.

## Author summary

*Trypanosoma brucei*, the parasite responsible for sleeping sickness in humans, is transmitted by the tsetse fly that primarily uses amino acids for its energy production. In the glucose-free environment encountered between the insect blood meals, *T*. *brucei* needs to produce through gluconeogenesis glucose 6-phosphate, a key precursor for several essential metabolic pathways. We have shown here that two key gluconeogenic steps, which produce phosphoenolpyruvate and fructose 6-phosphate, respectively, are performed by redundant enzymes (PPDK and PEPCK for phosphoenolpyruvate production; FBPase and a yet unknown enzyme for fructose 6-phosphate production), which highlights the importance of this metabolic pathway for the insect stages of the parasite. Interestingly, deletion of the parasite *FBPase* gene abolished both the colonisation of the insect salivary glands and the *in vitro* differentiation of the epimastigote forms into the mammalian infective form of the parasite. Altogether, these data demonstrate for the first time that gluconeogenesis is essential for development of *T*. *brucei* in its insect vector and that early development stages of the parasite present in the tsetse midgut are not affected by the absence of FBPase, probably by developing an alternative yet unknown approach to produce fructose 6-phosphate.

## Introduction

Trypanosomes of the *Trypanosoma brucei* species complex are the etiological agents of Human African Trypanosomiasis, a parasitic disease that affects about 20 countries in sub-Saharan Africa [1]. *T*. *brucei* adapts to the different environments encountered in its insect (tsetse fly) and mammalian hosts by remodeling its metabolism. In the glucose-rich environment of mammalian blood, the bloodstream forms of *T*. *brucei* rely solely on glucose to produce energy. However, in the glucose-free midgut environment of its insect vector, the procyclic form (PCF) of the parasite develops an elaborated energy metabolism based on amino acids such as proline, which has recently been proved to be essential for the parasite development, at least in the tsetse midgut [2, 3].

**Fig 1:**
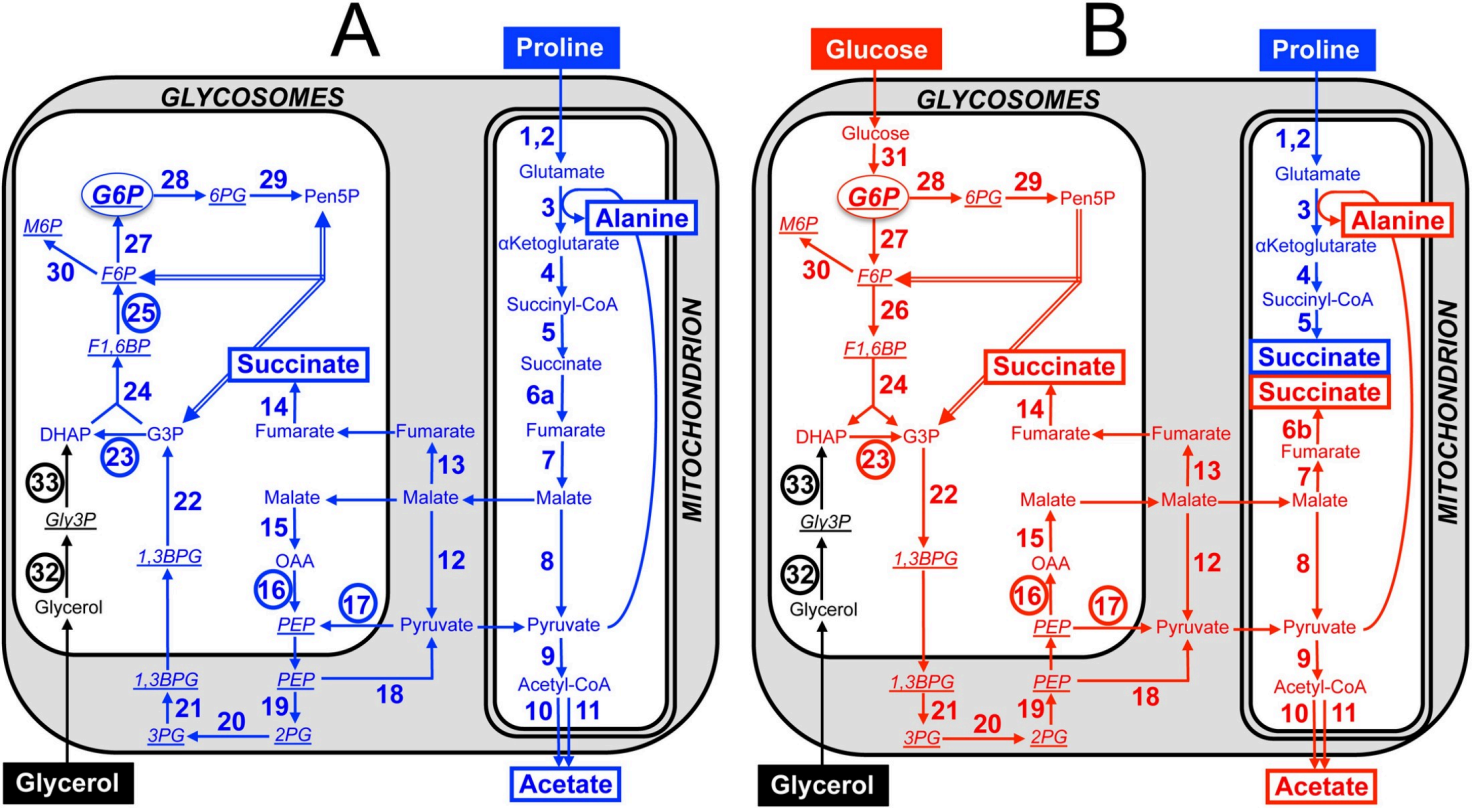
Proline, glucose and glycerol metabolism of the PCF trypanosomes. Panel A shows a schematic representation of the proline metabolism (blue) in PCF grown in glucose-depleted medium, with glycerol contribution possibly present in the medium (black for the glycerol-specific steps). Panel B corresponds to cells incubated in glucose-rich medium, with contribution of glucose to central carbon metabolism indicated in red. End products excreted from catabolism of proline and glucose are shown in a rectangle, the number corresponding to enzymes under investigation are circled and metabolites analysed by IC-MS/MS are underlined and in italic. For the sake of clarity, the reversible non-oxidative branch of the pentose phosphate pathway (PPP) is represented by double lines and neither the glycerol 3-phosphate (Gly3P)/dihydroxyacetone phosphate (DHAP) shuttle nor the cofactors and nucleotides are shown. Abbreviations: DHAP, dihydroxyacetone phosphate; F1,6BP, fructose 1,6-bisphosphate; 1,3BPG, 1,3-bisphosphoglycerate; F6P, fructose 6-phosphate; G3P, glyceraldehyde 3-phosphate; G6P, glucose 6-phosphate; Gly3P, glycerol 3-phosphate; 6PG, 6-phosphogluconolactone; M6P, mannose 6-phosphate; OAA, oxaloacetate; PEP, phosphoenolpyruvate; 2PG, 2-phosphoglycerate; 3PG, 3-phosphoglycerate; Pen5P, pentose 5-phosphate (ribulose 5-phosphate, ribose 5-phosphate and xylose 5-phosphate). Indicated enzymes are: 1, proline dehydrogenase; 2, pyrroline-5 carboxylate dehydrogenase; 3, L-alanine aminotransferase; 4, α-ketoglutarate dehydrogenase complex; 5, succinyl-CoA synthetase; 6a, succinate dehydrogenase (complex II of the respiratory chain); 6b, mitochondrial NADH-dependent fumarate reductase; 7, mitochondrial fumarase; 8, mitochondrial malic enzyme; 9, pyruvate dehydrogenase complex; 10, acetate:succinate CoA-transferase (ASCT); 11, acetyl-CoA thioesterase; 12, cytosolic malic enzyme; 13, cytosolic fumarase; 14, glycosomal NADH-dependent fumarate reductase; 15, glycosomal malate dehydrogenase; 16, phosphoenolpyruvate carboxykinase (PEPCK); 17, pyruvate phosphate dikinase (PPDK); 18, pyruvate kinase; 19, enolase (ENO); 20, phosphoglycerate mutase; 21, cytosolic phosphoglycerate kinase; 22, glyceraldehyde-3-phosphate dehydrogenase; 23, triose-phosphate isomerase (TIM); 24, aldolase; 25, fructose-1,6-bisphosphatase (FBPase); 26, phosphofructokinase; 27, glucose-6-phosphate isomerase; 28, glucose-6-phosphate dehydrogenase (G6PDH); 29, 6-phosphogluconolactonase, 6-phosphogluconate dehydrogenase, ribose-5-phosphate isomerase and ribulose-5-phosphate epimerase; 30, phosphomannose isomerase; 31, hexokinase; 32, glycerol kinase (GK); 33, NADH-dependent glycerol-3-phosphate dehydrogenase (GPDH).

Although glucose is absent from the tsetse midgut lumen between blood meals, the PCF of *T*. *brucei* prefers glucose to proline when both carbon sources are available [4]. In these conditions, glucose is converted by aerobic fermentation to the partially oxidised and excreted end products, succinate and acetate [5, 6]. The first seven steps of glycolysis are sequestered within peroxisome-like organelles, called glycosomes [7, 8]. Phosphoenolpyruvate (PEP) is produced in the cytosol, where it is located at a branching point to feed the glycosomal ‘succinate branch’ and the mitochondrial ‘acetate and succinate branches’ (Fig 1B). Both succinate branches are initiated by the glycosomal PEP carboxykinase (PEPCK, EC 4.1.1.49, step 16 in Fig 1) by conversion of PEP into oxaloacetate, which is further converted into malate in the glycosomes (step 15), before being metabolised into succinate in both the glycosomes (steps 13 and 14) and the mitochondrion (steps 6B and 7 in Fig 1B) [9, 10]. PEPCK, together with the glycosomal pyruvate phosphate dikinase (PPDK, EC 2.7.9.1, step 17) [11], are directly involved in the maintenance of the glycosomal ADP/ATP balance, by regenerating the ATP consumed by the first and third glycolytic steps (hexokinase, EC 2.7.1.1, step 31 and phosphofructokinase, PFK, EC 2.7.1.11, step 26,) [12]. Part of PEP is also converted in the cytosol to pyruvate, which enters the mitochondrion to feed the pyruvate dehydrogenase complex (PDH, EC 1.2.4.1, step 9) for acetyl-CoA production, which is further converted into excreted acetate by mitochondrial acetate:succinate CoA-transferase (ASCT, EC 2.8.3.8, step 10) and acetyl-CoA thioesterase (ACH, EC 3.1.2.1, step 11) [13–15]. It is noteworthy that a canonical tricarboxylic acid cycle, with acetyl-CoA being converted into CO_2_, is not operative in trypanosomes [16]. In glucose-rich condition, proline contributes moderately to central carbon metabolism and is primarily converted into the excreted end product succinate (Fig 1B) [4].

In contrast, when the PCF are incubated in the absence of glucose, a situation likely encountered by trypanosomes in their different *in vivo* niches, proline becomes the main carbon source used by the parasite, being consumed up to 6-fold more [4]. In these conditions, proline feeds the whole central carbon and energy metabolism (Fig 1A). For instance, proline-derived succinate produced in the mitochondrion, even in the presence of glucose (steps 1–5 in Fig 1B), is further metabolised and converted into excreted alanine [4]. The absence of glucose also implies that glucose 6-phosphate (G6P), a precursor for essential pathways, such as the pentose phosphate pathway (PPP) and nucleotide sugar biosynthesis [17, 18], needs to be produced by reversal of glycolysis through the so-called gluconeogenesis. Under physiological conditions, all glycolytic enzymes involved in G6P conversion into PEP catalyse a reversible reaction used for glyconeogenesis, except PFK that is replaced by fructose-1,6-bisphosphatase (FBPase, EC 2.7.1.40, step 25), as observed in many organisms including *Leishmania major* [19]. In addition, the production of the main gluconeogenic precursor, PEP, can theoretically be performed in trypanosomatids by PPDK from pyruvate and/or by PEPCK from oxaloacetate. In the promastigote form of *L*. *major*, PPDK and PEPCK both contribute to mannogen biosynthesis through gluconeogenesis from alanine and aspartate, respectively [20]. In contrast, proline has been reported to be incorporated into hexose phosphates through gluconeogenesis in the PCF of *T*. *brucei* grown in the absence of glucose, however the contributions of PPDK and PEPCK in these conditions have not been addressed so far [21].

Here, we used a combination of multiple mutations (up to three genes targeted at the same time by knock-out and/or RNAi-mediated knock-down) and metabolomic analyses to study the role of the key gluconeogenic enzymes in the PCF trypanosomes. We showed that PPDK and PEPCK have a redundant essential function for the incorporation of [^13^C]-enriched proline into gluconeogenic intermediates. In addition, gluconeogenesis from glycerol is abolished in the glycerol kinase (GK, EC 2.7.1.30, step 32) null background. *FBPase* gene knock-out abolished colonisation of the tsetse fly vector salivary glands by the parasite, as well as *in vitro* differentiation of the epimastigote forms into the metacyclic form, demonstrating the crucial role of gluconeogenesis, at least during the last part of the trypanosome cyclical development in the tsetse fly.

## Results

### PPDK and PEPCK are essential for gluconeogenesis from proline

**Fig 2:**
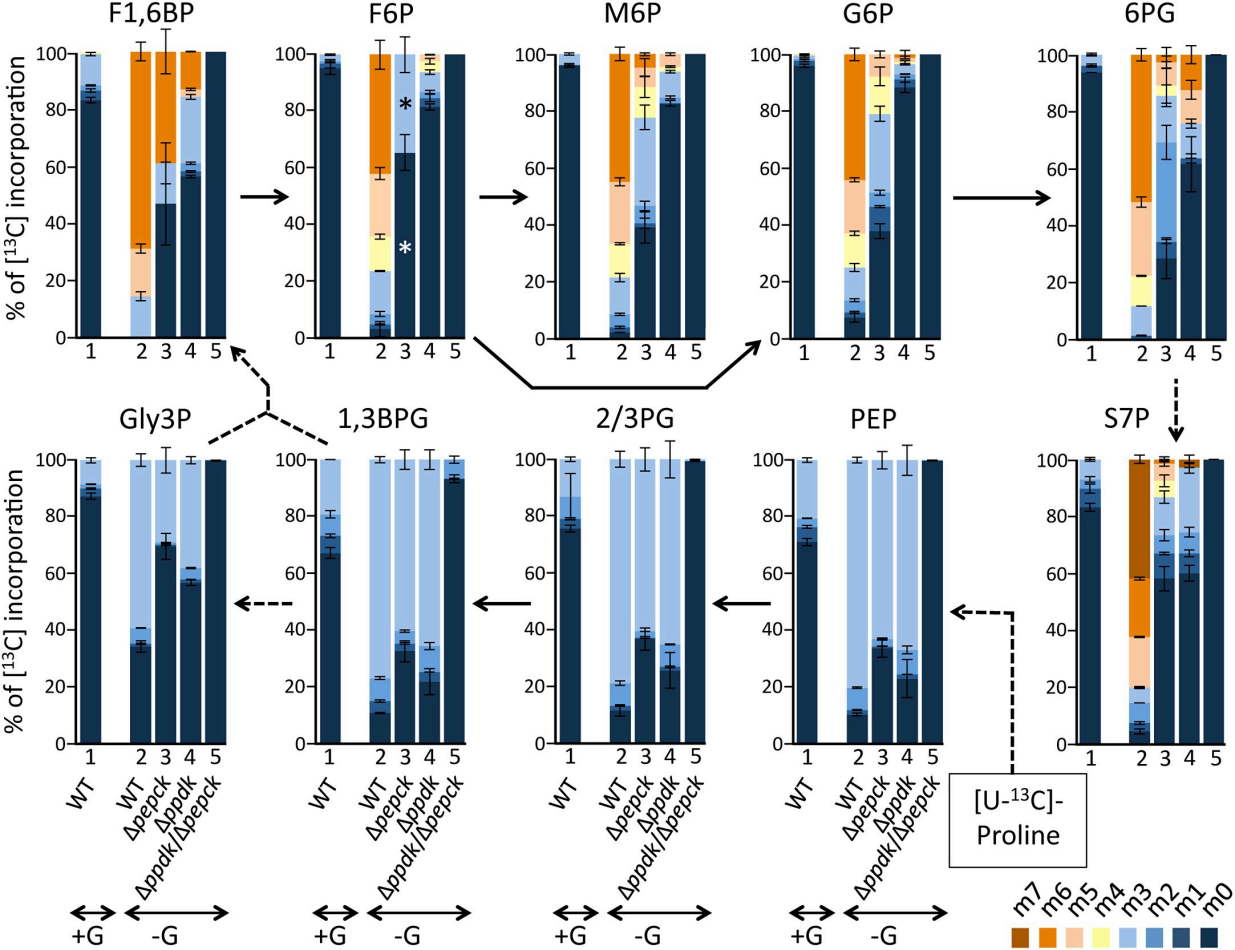
IC-MS/MS analysis of intracellular metabolites after isotopic labelling with [U-^13^C]-proline. The EATRO1125.T7T parental (WT), Δ*pepck*, Δ*ppdk* and Δ*ppdk*/Δ*pepck* cell lines were incubated for 2 h in PBS containing 2 mM [U-^13^C]-proline with 2 mM glucose (+G, only the parental cells) or without glucose (-G) prior to metabolite extraction. The figure shows enrichment of key glycolytic intermediates at 0 to 6 carbon positions (m0 to m6) with ^13^C expressed as percentage of all corresponding molecules (MID; Mass Isotopomer Distribution). For abbreviations, see Fig 1: S7P, sedoheptulose 7-phosphate; 2/3PG means that 2-phosphoglycerate (2PG) and 3-phosphoglycerate (3PG) are undistinguished by IC-MS/MS. The asterisks (*) in the F6P column for the Δ*pepck* mutant highlights that the absolute amount of this metabolite being particularly low in this incubation conditions, solely the m0 and m3 isotopomer were detected.

PCF trypanosomes incubated in glucose-free and proline-rich conditions are able to maintain significant levels of (glycolytic) sugar-phosphates, thought at lower extent than cells incubated with glucose and proline (S1 Table). This means that proline is able to support the synthesis of the sugar-phosphates by a gluconeogenesis process. According to the current view of the central carbon metabolism of the PCF trypanosomes, PPDK and PEPCK are the only enzymatic steps that can produce PEP, the precursor of gluconeogenesis, in proline catabolism (steps 16 and 17 in Fig 1A). These two enzymes have been shown to contribute to glucose catabolism, however, their roles in glucose-depleted conditions have not been investigated so far [12, 22]. To investigate this poorly explored gluconeogenic metabolic pathway from proline, we have determined by mass spectrometry the incorporation levels of ^13^C atoms from uniformly [^13^C]-enriched proline ([U-^13^C]-proline) into key gluconeogenic intermediates of parental and knock-out (Δ*ppdk*, Δ*pepck* and Δ*ppdk*/Δ*pepck*) PCF cell lines. Cells were incubated in PBS containing 2 mM [U-^13^C]-proline in the presence or absence of the same amounts of non-labelled glucose. Incorporation of ^13^C into glycolytic intermediates was quantified by IC-MS/MS and the values for selected glycolytic metabolites are shown in Fig 2. Incorporation of ^13^C atoms into hexose phosphate glycolytic intermediates from [U-^13^C]-proline is very low in the presence of glucose (3.1% on average), compared to cells incubated in the absence of glucose (94.1% on average). The same labelling pattern was also observed for the intermediates of the pentose phosphate pathways, *i*.*e*. 6-phosphogluconolactone and sedoheptulose 7-phosphate. This confirms that proline feeds gluconeogenesis and the pentose phosphate pathway in the absence of glucose, while the presence of glucose down-regulates conversion of proline into metabolite intermediates of these pathways, as we previously observed [21]. Incorporation of at least two ^13^C atoms into the same hexose phosphates is more reduced in the Δ*ppdk* mutant than in the Δ*pepck* mutant (13.8% *versus* 49.3% on average, respectively), suggesting that PPDK might contribute more than PEPCK to gluconeogenesis from proline (Fig 2). More importantly, this ^13^C-incorporation is abolished in the Δ*ppdk*/Δ*pepck* double mutant (not detectable in Fig 2), although proline consumption is increased by 2-fold [12, 21]. This demonstrates that PPDK and PEPCK are the only enzymes significantly contributing to PEP production from proline catabolism and have a redundant function for the essential production of glycolytic intermediates from proline. These data also confirm that, under the conditions pertaining in the cell, pyruvate kinase (EC 2.7.1.40, step 18 in Fig 1) irreversibly converts PEP into pyruvate, even in the absence of PEPCK and PPDK.

### Glycerol is an alternative gluconeogenic carbon source

**Fig 3:**
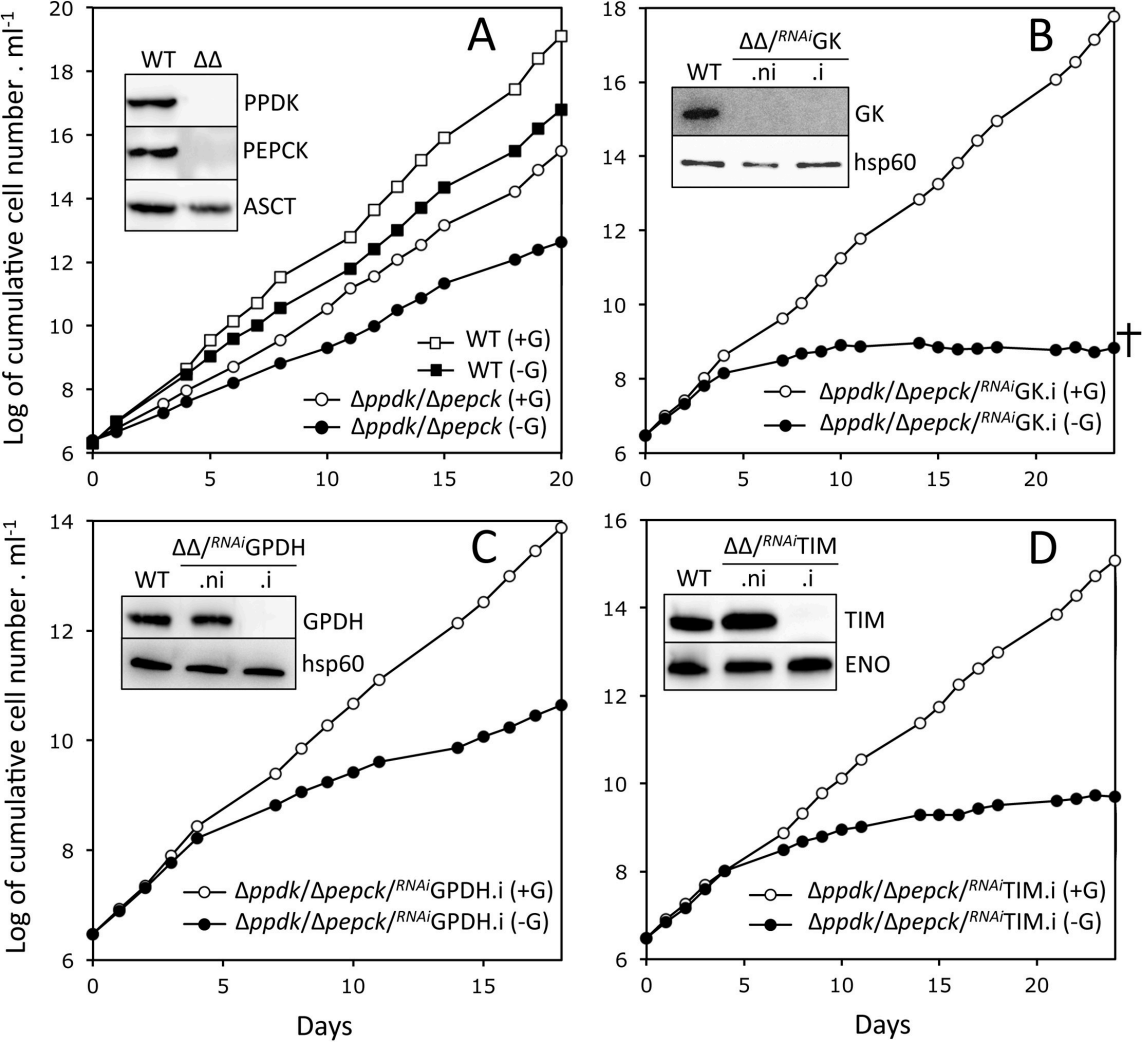
Functional analysis of Δ*ppdk*/Δ*pepck* cell lines. This figure represents growth curves of the Δ*ppdk*/Δ*pepck* cell line (panel A) and tetracycline-induced Δ*ppdk*/Δ*pepck*/^*RNAi*^GK.i, Δ*ppdk*/Δ*pepck*/^*RNAi*^GPDH.i and Δ*ppdk*/Δ*pepck*/^*RNAi*^TIM.i triple mutants (panels B-D) grown in SDM79-GlcFree medium containing 10 mM glucose (+G) or not (-G). In glucose-free conditions, the SDM79-GlcFree medium was supplemented with 50 mM *N*-acetyl-D-glucosamine that inhibits uptake of residual glucose. Cells were maintained in the exponential growth phase (between 10^6^ and 10^7^ cells ml^-1^). The insets show western blot analyses of the parental (WT), Δ*ppdk*/Δ*pepck* (ΔΔ) and tetracycline-induced (.i) and non-induced (.ni) triple mutants with the immune sera indicated in the right margin (ASCT, acetate:succinate CoA-transferase; ENO, enolase). The cross in panel B means that the mutant ultimately dies.

To explore whether blocking gluconeogenesis from proline affects parasite growth, we developed an SDM79-derived glucose-free medium (SDM79-GlcFree) containing less than 1 μM glucose, as determined by NMR spectrometry and supplemented with 50 mM *N*-acetyl D-glucosamine, a competitive inhibitor of glucose transport [21]. The growth of the parental EATRO1125.T7T PCF cell line is moderately affected by the absence of glucose with a doubling time increased by ~20% in the SDM79-GlcFree compared to the same medium supplemented with 10 mM glucose (Fig 3A). Similarly, growth of the Δ*ppdk*/Δ*pepck* cell line is not compromised in the absence of glucose, suggesting that another gluconeogenic carbon source can be used by the PCF in addition to proline (Fig 3A). The abolition of PEP production from gluconeogenic amino acids (including proline), as demonstrated above, strongly supports the view that glycerol might be a possible alternative to proline that would feed gluconeogenesis in the Δ*ppdk*/Δ*pepck* cell line. To address this hypothesis, we have measured by IC-MS/MS the [^13^C]-incorporation into glycolytic intermediates of the parental PCF incubated with [U-^13^C]-glycerol. In this experiment, most hexose phosphate glycolytic intermediates (83.2% on average) are fully [^13^C]-enriched after 2 h of incubation with [U-^13^C]-glycerol as the only carbon source (S1 Fig).

### Blocking gluconeogenesis from both proline and glycerol affects growth of the PCF trypanosomes in glucose-free conditions

In order to abolish all possible gluconeogenic pathways, the expression of the key enzymes of glycerol conversion pathway was successively down-regulated in the Δ*ppdk*/Δ*pepck* null background, *i*.*e*. glycerol kinase (GK, EC 2.7.1.30, Tb927.9.12550-Tb927.9.12630, step 32), glycerol-3-phosphate dehydrogenase (GPDH, EC 1.1.1.8, Tb927.8.3530, step 33) and triose phosphate isomerase (TIM, EC 5.3.1.1, Tb927.11.5520, step 23). We hypothesised that the abolition of gluconeogenesis from both proline and glycerol, in the Δ*ppdk*/Δ*pepck*/^*RNAi*^GK, Δ*ppdk*/Δ*pepck*/^*RNAi*^GPDH and Δ*ppdk*/Δ*pepck*/^*RNAi*^TIM triple mutants, should lead to the death of parasites grown in glucose-free conditions. Indeed, the growth of the tetracycline-induced Δ*ppdk*/Δ*pepck*/^*RNAi*^GK cell line (Δ*ppdk*/Δ*pepck*/^*RNAi*^GK.i) in SDM79-GlcFree medium stopped after 10 days of tetracycline induction and cells ultimately died two weeks later, while the addition of glucose restored their growth (Fig 3B). Similarly, the growth of the Δ*ppdk*/Δ*pepck*/^*RNAi*^TIM.i mutant was strongly affected by the absence of glucose, but this was not lethal to the cells (Fig 3D). However, the growth of the Δ*ppdk*/Δ*pepck*/^*RNAi*^GPDH.i cells appears to be only moderately affected by the absence of glucose, although GPDH was not detected by western blotting anymore (Fig 3C), probably because the glycerol 3-phosphate/dihydroxyacetone phosphate shuttle could bypass the GPDH step (see Fig 2 in [5] for this pathway).

**Fig 4:**
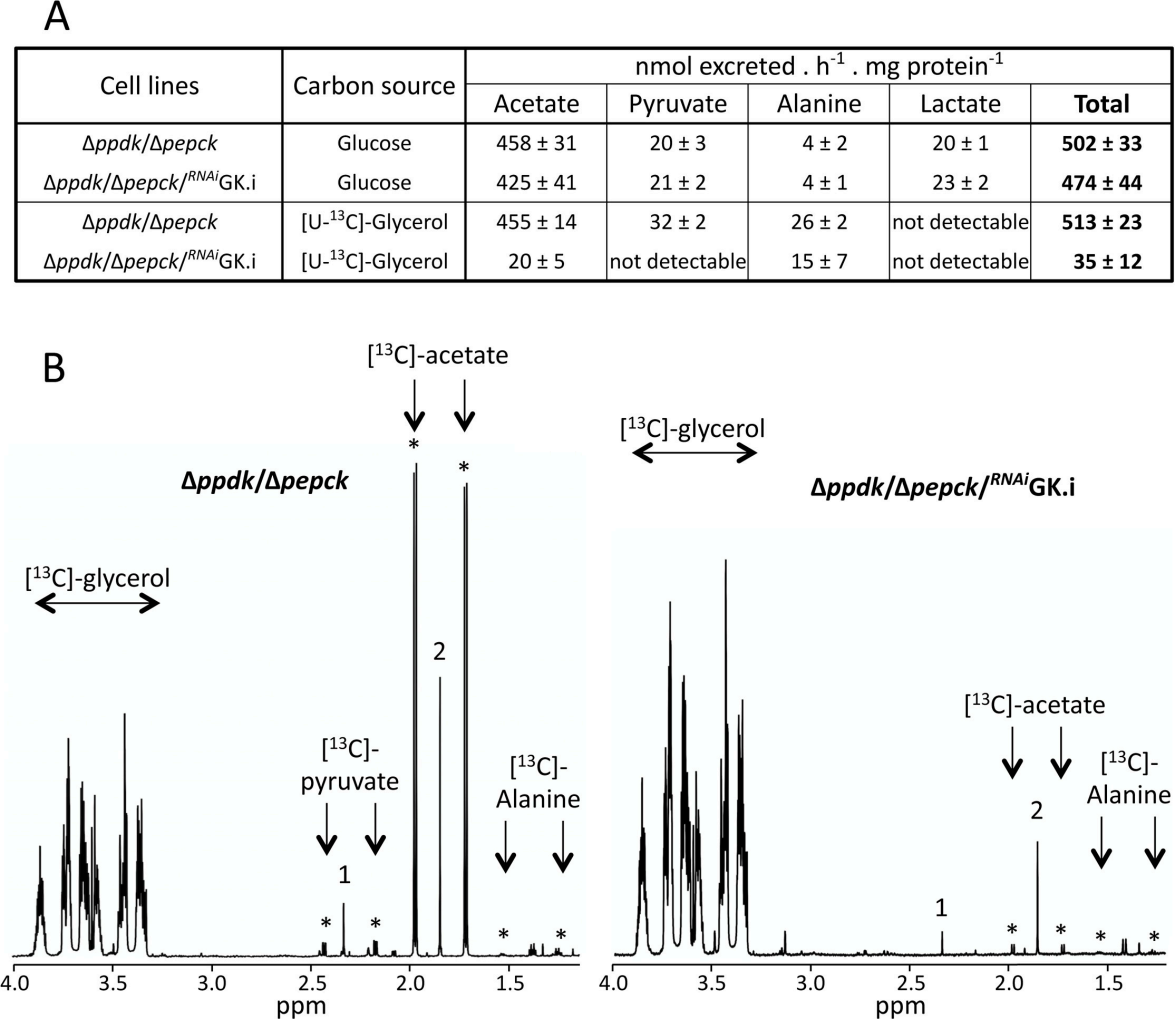
Proton (^1^H) NMR analysis of end products excreted from glucose and [U-^13^C]-glycerol metabolism. The Δ*ppdk*/Δ*pepck* and tetracycline-induced (.i) Δ*ppdk*/Δ*pepck*/^*RNAi*^GK cell lines were incubated for 6 h in PBS/NaHCO_3_ buffer containing 4 mM glucose or 4 mM [U-^13^C]-glycerol. Panel A shows the quantitative data of excreted end products from glucose or [U-^13^C]-glycerol metabolism expressed as nmol of acetate, pyruvate, alanine and lactate excreted per h and per mg of protein. Panel B shows one representative NMR spectrum (out of 3 spectra and ranging from 1.2 to 4 ppm) of end products excreted from [U-^13^C]-glycerol metabolism of the Δ*ppdk*/Δ*pepck* and Δ*ppdk*/Δ*pepck*/^*RNAi*^GK.i cell lines. The resonance massif corresponding to unconsumed [U-^13^C]-glycerol as indicated (from 3.3 to 3.8 ppm) and resonances indicated by asterisks correspond to [^13^C]-enriched pyruvate and acetate. The resonances labelled 1 and 2 correspond to non-[^13^C]-enriched succinate and acetate, respectively, produced from the catabolism of an unknown internal carbon source [6, 23].

To determine whether the glycerol metabolism is abolished in the Δ*ppdk*/Δ*pepck*/^*RNAi*^GK.i cell line that shows the strongest growth alteration in SDM79-GlcFree medium, quantitative analyses of ^13^C-enriched end products excreted from [U-^13^C]-glycerol metabolism were performed by proton NMR spectrometry [6]. It is noteworthy that (*i*) the use of ^13^C-enriched glycerol as carbon source allows us to distinguish between the products excreted from metabolism of [U-^13^C]-glycerol and an uncharacterised internal carbon source [6, 23], and (*ii*) the succinate excretion is abolished in the Δ*ppdk*/Δ*pepck*/^*RNAi*^GK.i mutant (Fig 4), as previously observed for the Δ*ppdk*/Δ*pepck* and Δ*pepck* cell lines [12, 22], since the succinate branch is interrupted in the absence of PEPCK (see Fig 1B). The Δ*ppdk*/Δ*pepck*/^*RNAi*^GK.i mutant shows a 15-fold decrease of the total end products excreted from glycerol breakdown (acetate, alanine, pyruvate and lactate) compared to the parental Δ*ppdk*/Δ*pepck* mutant, while glucose metabolism is not affected (Fig 4). This confirms that the growth arrest observed for the Δ*ppdk*/Δ*pepck*/^*RNAi*^GK.i is due to the abolition of glycerol metabolism that is required to feed gluconeogenesis in the PPDK/PEPCK null background in glucose-free conditions.

### FBPase is not essential for PCF trypanosomes in glucose-free *in vitro* conditions

**Fig 5:**
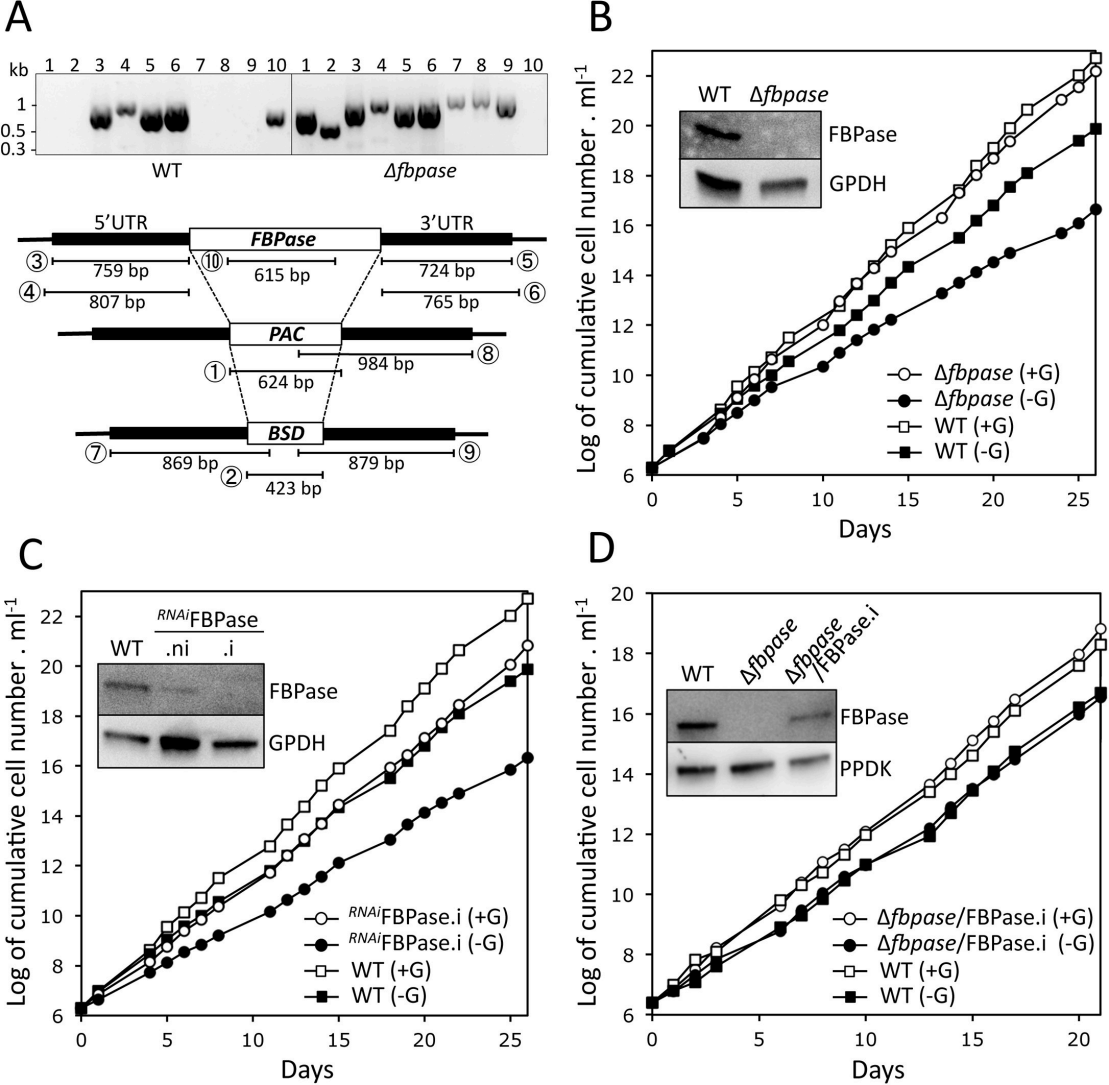
Analysis of FBPase mutants cell lines. Panel A shows a PCR analysis of genomic DNA isolated from the parental (WT) and Δ*fbpase* cell lines. The 1 to 10 lanes of the gel picture correspond to different PCR products described in the lower panel with circled numbers. As expected, PCR amplification of the *FBPase* gene (lane 10) was only observed in the parental cell line, while *BSD* and *PAC* PCR-products were observed only in the Δ*fbpase* mutant (lanes 1–2 and 7–9). As control, both the 5'- and 3'-untranslated regions (UTRs) are PCR amplified from the parental and Δ*fbpase* samples (lanes 3–6). Panels B-D show the growth curves of the Δ*fbpase* (B), ^*RNAi*^FPBase.i (C) and Δ*fbpase/*FPBase.i (D) mutant cell lines, together with the parental (WT) cell line, incubated in SDM79-GlcFree medium containing 10 mM glucose (+G) or not (-G). Western blot analyses with the immune sera indicated in the right margin of the parental (WT), Δ*fbpase*, the tetracycline-induced (.i) and non-induced (.ni) ^*RNAi*^FPBase and/or Δ*fbpase/*FPBase.i mutant are shown in the insets.

Gluconeogenesis from glycerol and proline leads to the production of fructose 1,6-bisphosphate (F1,6BP), that is converted into fructose 6-phosphate (F6P) by the fructose-1,6-bisphosphatase (FBPase, EC 3.1.3.11, step 25 in Fig 1A), a well-known gluconeogenic enzyme that is, however, not involved in glycolysis. To confirm the gluconeogenic role of FBPase in PCF, both alleles of the single copy *FBPase* gene (Tb927.9.8720) were replaced by the blasticidin and puromycin markers (Δ*fbpase*) and FBPase expression was conditionally down-regulated by RNAi (^*RNAi*^FBPase). Both the Δ*fbpase* and ^*RNAi*^FBPase.i cell lines are viable in the absence of glucose, although with a similarly increased doubling time compared to the parental cells (18.4 h and 18.5 h, respectively, compared to 13.7 h) (Fig 5B and 5C). These data suggest that, although the absence of FBPase affects parasite growth in glucose-free conditions, an alternative enzyme or pathway could substitute for the FBPase reaction, in such a way that proline and/or glycerol would contribute to G6P production even in the absence of FBPase. Re-expression of a rescue ectopic copy of the *FBPase* gene in the Δ*fbpase* background using the tetracycline-inducible pLew100 vector (Δ*fbpase*/FBPase.i cell line) restored the WT growth (Fig 5D).

**Fig 6:**
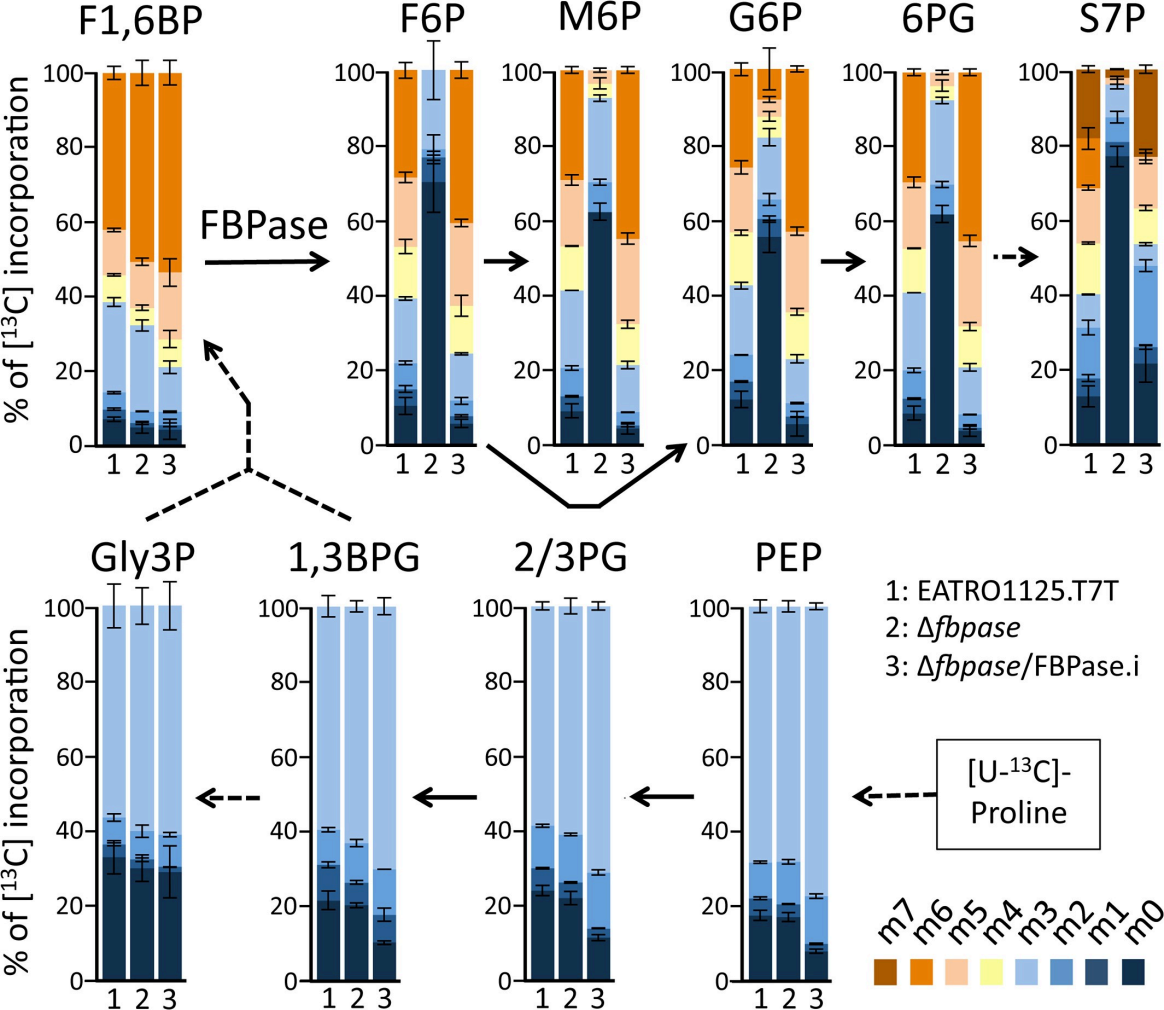
IC-MS/MS analysis of intracellular metabolites of the Δ*fbpase* mutant incubated with [U-^13^C]-proline. The parental (EATRO1125.T7T), Δ*fbpase* and Δ*fbpase*/FBPase.i cell lines were incubated for 2 h in PBS containing 2 mM [U-^13^C]-proline. Enrichment of key glycolytic intermediates at carbon positions 0 to 6 (m0 to m6) is expressed as percentage of all the corresponding molecules. For abbreviations, see Figs 1 and 2.

To confirm the role of FBPase in gluconeogenesis, we determined by IC-MS/MS the incorporation levels of [U-^13^C]-proline into glycolytic intermediates of the WT, Δ*fbpase* and Δ*fbpase*/FBPase.i rescue cell lines (Fig 6). The incorporations of [^13^C]-enriched atoms from proline into triose phosphates (PEP, 2/3PG, 1,3BPG and Gly3P, see Fig 1 for abbreviations) and F1,6BP are equivalent in the parental, Δ*fbpase* and Δ*fbpase*/FBPase.i cell lines. However, the incorporations of [^13^C]-enriched atoms in F6P (the product of the reaction catalysed by FBPase), as well as in metabolites produced from F6P, *i*.*e*. G6P, mannose 6-phosphate (M6P) and 6-phosphogluconolactone (6PG) and sedoheptulose 7-phosphate (S7P), are strongly reduced in the Δ*fbpase* mutant compared to the parental cell line. Indeed, the relative amounts of non-enriched F6P, G6P, M6P and 6PG and S7P in the Δ*fbpase* cells are increased by 6.5-, 4.6-, 6.9, 7.2- and 5.8-fold, respectively. As expected, incorporation of ^13^C-carbons into hexose phosphates is restored in the Δ*fbpase*/FBPase.i rescue cell line (Fig 6). These data clearly demonstrate that gluconeogenesis from proline, although strongly reduced, still occurs in the absence of FBPase, which implies the existence of an alternative step to FBPase producing G6P from triose phosphates.

**Fig 7:**
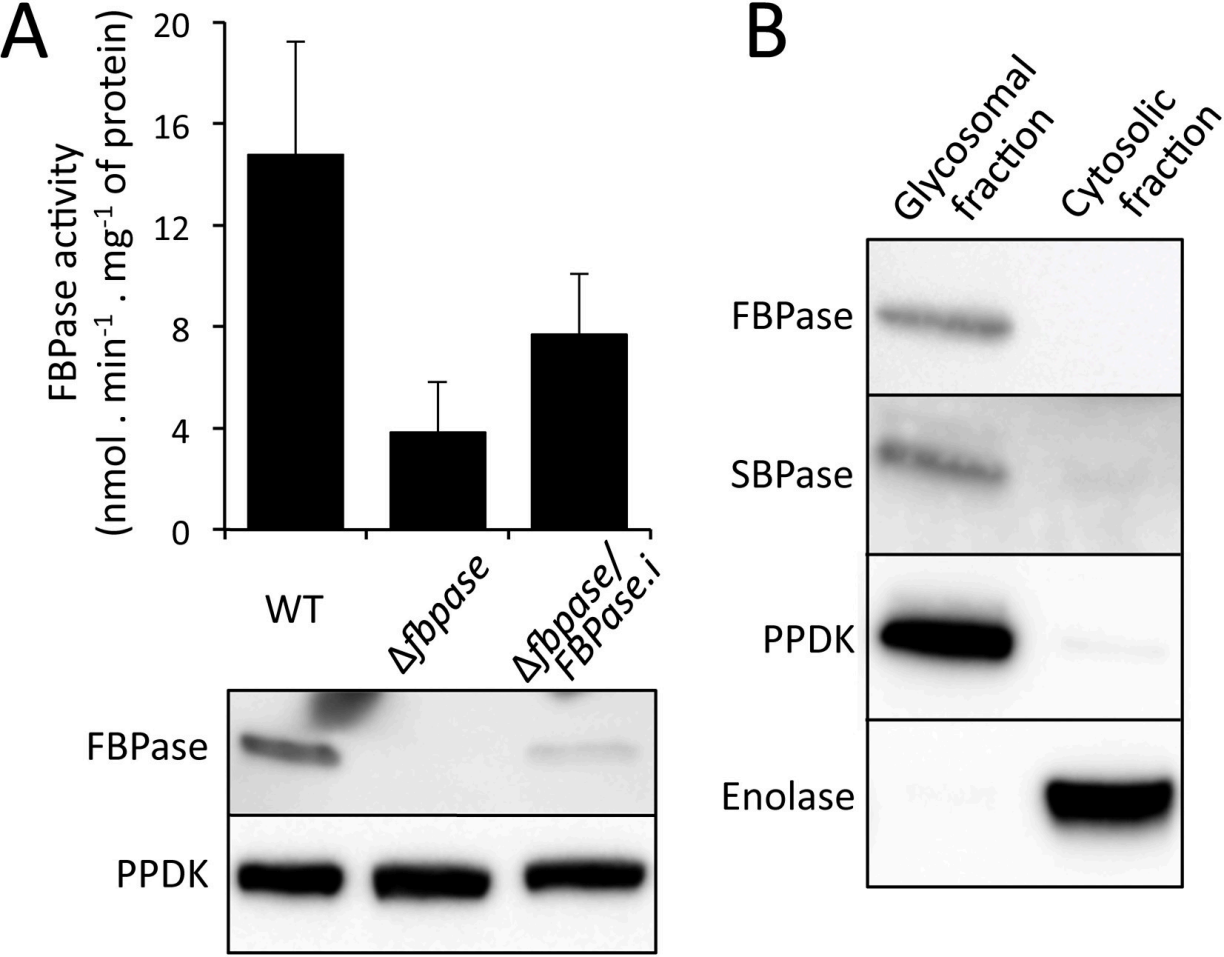
The glycosomal FBPase activity is not abolished in the Δ*fbpase* mutant. Panel A shows the FBPase activity (top panel) and western blotting analysis (lower panel) of glycosomal fractions of the parental (WT), Δ*fbpase* and Δ*fbpase*/FBPase.i (rescue) cell lines. The FBPase activities were normalised with the glycerol kinase activities. In panel B the glycosomal localisation of FBPase and SBPase was confirmed by western blotting analyses on glycosomal and cytosolic fractions, using control immune sera against glycosomal (PPDK) and cytosolic (enolase) markers.

To order to highlight the alternative to FBPase, the FBPase activity was determined on the parental, Δ*fbpase* and Δ*fbpase*/FBPase.i (rescue) cell lines. This enzymatic activity was not detectable on total cellular extracts, but was detectable in the glycosomal fractions of the parental cell line at a low level compared to the glycerol kinase activity (14.8 ±4.5 *versus* 11,100 ±2,300 nmol min^-1^ mg^-1^ of proteins) (Fig 7A). Interestingly, this activity is not abolished in the Δ*fbpase* mutant, although decreased by 3.8-fold. Reintroduction of the *FBPase* gene in the Δ*fbpase* cell line only partially restored the FBPase activity (7.1 ±2.3 *versus* 14.8 ±4.5 nmol min^-1^ mg^-1^ of proteins), which is consistent with the lower level of FBPase expression in the rescue cell line compared to that of the parental cells (Fig 7A). Altogether, these data strongly support the view that an unknown glycosomal enzyme is responsible for approximately one fourth of the glycosomal FBPase activity. Incidentally, the *T*. *brucei* genome contains a single gene potentially coding for a sedoheptulose-1,7-bisphosphatase (SBPase, Tb927.2.5800), which belongs to the same superfamily as the FBPases. Western blot analysis of the glycocomal and cytosolic fractions of the parental trypanosomes with the anti-FBPase and anti-SBPase immune sera demonstrated the glycosomal localisation of these two proteins (Fig 7B), which is in agreement with previously published proteomic analyses of glycosomal fractions [24, 25].

### FBPase is essential for the establishment of mature infections in the insect fly salivary glands

**Fig 8:**
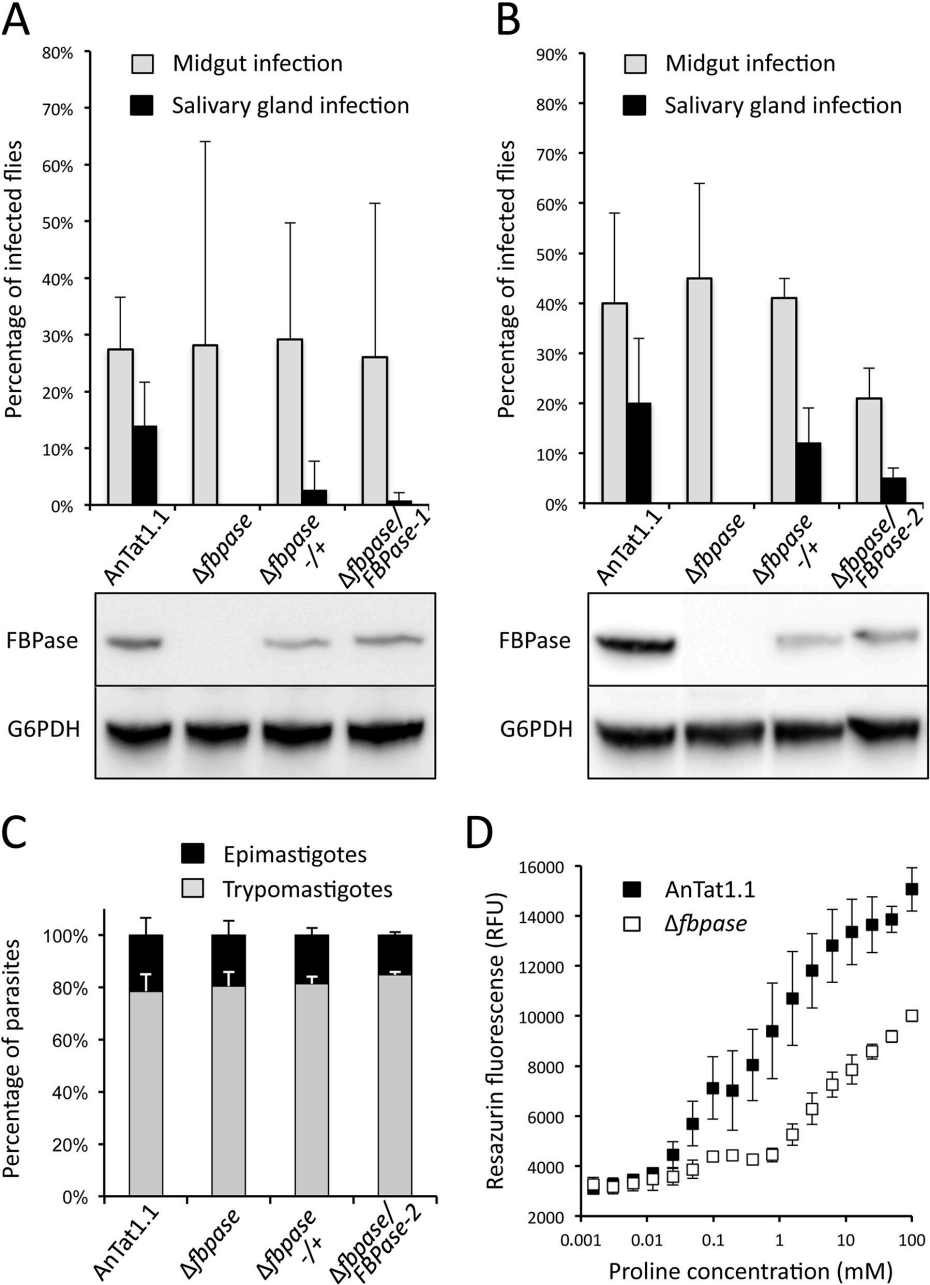
The deletion of the *FBPase* gene prevents parasites to colonise the tsetse salivary glands. In this experiment, batches of 50 *Glossina morsitans morsitans* teneral males were artificially fed with either the parental AnTat1.1E wild-type, the Δ*fbpase*-/+ (one allele deleted), the Δ*fbpase* (null mutant) or the Δ*fbpase*/FBPase-1 and -2 (rescue cell lines) PCF cell lines in culture medium as previously described [28]. Four weeks after the infective meal, a total of 790 flies were dissected to assess the presence of parasites in their midgut and salivary glands by microscopic examination. Midgut and salivary gland infection rates (in % ±SD) are presented for each cell line as the mean of 4 independent biological replicates done with the rescue cell line expressing the FBPase through the pLew100 vector (panel A), and of 6 independent biological replicates done with the rescue cell line expressing the FBPase from an ectopic *FBPase* copy in the *FBPase* locus (panel B). The proportion of flies developing a salivary gland infection are: 12/96 for the parental AnTat1.1E, 0/60 for Δ*fbpase*, 3/104 for Δ*fbpase*-/+ and 1/84 for Δ*fbpase*/FBPase-1 in panel A and 33/163 for the parental AnTat1.1E, 0/91 for Δ*fbpase*, 8/65 for Δ*fbpase*-/+ and 6/125 for Δ*fbpase*/FBPase-2 in panel B. Below the graphs are shown western blot analyses of the FBPase expression using the glucose-6-phosphate dehydrogenase (G6PDH) as loading control. (C) The proportions of cells in the different morphotypes found in the proventriculus and midgut were compared after dissection of flies infected with either one the four different cell lines from at least two independent biological replicates. The proportion of trypomastigotes and epimastigotes is indicated in grey and black respectively. 692, 373, 432, and 280, cells isolated from 6, 2, 3 and 2 flies have been analysed for the parental AnTat1.1E, Δ*fbpase*, Δ*fbpase*-/+ and Δ*fbpase*/FBPase-2 cell lines, respectively. Panel D shows growth of the AnTat1.1E and Δ*fbpase* cell lines in the SDM79-GlcFree medium containing from less than 20 μM to 100 mM proline, using the Alamar Blue assay. The proline concentration values on the x axis correspond to the amounts of proline added to the proline-depleted SDM79-GlcFree medium, which contains less than 20 μM proline.

We then attempted to determine whether the absence of FBPase would affect parasite virulence *in vivo* in their tsetse fly vectors. To perform such experimental infections, a cell line bearing a single *FBPase* allele (Δ*fbpase*-/+), a FBPase null mutant (Δ*fbpase*), and two distinct constitutive add-back rescue cell lines (Δ*fbpase*/FBPase-1 and 2) were generated in the AnTat1.1E pleomorphic strain [26]. Indeed, this strain has maintained the ability to efficiently colonise salivary glands and to produce infectious metacyclic forms, as opposed to parasites with the EATRO1125 genetic background [27]. Two strategies were developed to produce the Δ*fbpase*/FBPase rescue cell lines, *i*.*e*. the addition of a *FBPase* ectopic copy under the control of the PARP promoter in the pLew100 vector (Δ*fbpase*/FBPase-1), and the *in-situ* re-insertion of one *FBPase* gene under the control of its endogenous UTRs (Δ*fbpase*/FBPase-2). Batches of about 50 teneral male tsetse flies were artificially fed through a silicone membrane with cultured PCF parasites of either one of the four cell lines in parallel (the parental wild-type, the Δ*fbpase*-/+, the Δ*fbpase* and the Δ*fbpase*/FBPase-1 or -2 cell lines). Flies were dissected after one month in order to quantify the infection rates per organ, the parasite densities per organ and the proportion of parasite morphotypes as previously described [28]. A total of 784 flies were dissected with similar results obtained with the two series: 338 flies out of 4 biological replicates with the Δ*fbpase*/FBPase-1 panel (Fig 8A) and 446 flies out of 6 replicates with the Δ*fbpase*/FBPase-2 panel (Fig 8B). First, the midgut infection rates were comparable in all conditions (33% on average, ranging from 19% ±27% in Fig 8A to 45% ±19% in Fig 8B for the null mutants). Second, although the salivary gland infection rates were on average 16% for the wild-type cells, 7% for the Δ*fbpase*-/+ parasites and 3% for the Δ*fbpase*/FBPase rescue cell line, no null mutant parasites were seen in any salivary glands and this observation was statistically significant (p = 0.011 by ANOVA Tukey add-hoc post-test at 95% confidence when comparing %SG+ in Δ*fbpase versus* wild-type). This demonstrates that FBPase is essential for the second part of *T*. *brucei* cyclical development, *i*.*e*. the establishment of mature infections in the insect salivary glands. The lower salivary gland infection rates observed with the Δ*fbpase*/FBPase cell lines could be explained by the additional round of transformation and the longer time spent in culture during the generation of this cell line that would both have affected its virulence. It is also noteworthy that the *in-situ* rescue approach is apparently more adapted than the use of the pLew100, probably because the PARP promoter used for driving gene expression in the pLew100 vector may be naturally less efficient in the short epimastigotes, attached epimastigotes and/or in the metacyclic stages during the late steps of the cyclical development as compared to the procyclic and mesocyclic midgut stages.

Assuming that (*i*) the parasite densities in the midgut, estimated by microscopic observations, were similar for all the cell lines and that (*ii*) no obvious motility defect was detected among these cell lines neither *in vitro* nor *in vivo*, we reasoned that the absence of FBPase may have impaired parasite differentiation, especially the first morphotype switch occurring in proventricular parasites. Therefore, parasites were isolated from the anterior midgut and proventriculus of infected flies, and stained with a DNA marker (DAPI) and an axonemal marker Mab25 [29] in order to determine the proportions of cells in each morphotype (trypomastigote *versus* epimastigote) according to the cell lines (Fig 8C). Nevertheless, no difference was detected between cell lines that were all displaying on average 80% of proventricular parasites in the trypomastigote morphotype and 20% of epimastigotes (280 to 692 cells out of 2 to 6 flies per cell line). This demonstrates again that FBPase is not essential for the trypanosome cyclical development in the midgut, while it is required at least for the initial colonisation of salivary glands.

We reasoned that these differences in behaviour could be the consequence of possible low proline concentrations in the oesophagus and/or the salivary glands of the tsetse, which would not be sufficient for feed gluconeogenesis in the absence of FBPase. To test this hypothesis, we estimated the growth of the parental and Δ*fbpase* cell lines as a function of proline concentrations in the SDM79-GlcFree medium, using the Alamar Blue assay. As expected these two cell lines failed to grow under less than 20 μM proline, this amino acid being the primary carbon source used by the parasite to feed its central carbon metabolism (Fig 8D). Growth of the parental cells is considerably improved by addition of at least 20 μM proline, the parental cells growing only 2-times slower in the presence of 0.5 mM compared to 5 mM proline (concentration in the SDM79-GlcFree medium). In contrast the Δ*fbpase* cells are barely growing in the presence of 0.5 mM proline. This analysis showed that the Δ*fbpase* mutant requires ~30-times more proline to grow at the same speed as the parental cells (Fig 8D), which may provide a rational explanation of the observed difference in behaviour of these two cell lines *in vivo* if proline concentrations are higher in the midgut compared to the oesophagus and/or the salivary glands. Unfortunately, the concentrations of proline in the tsetse organs are unknown. It is noteworthy that, immunofluorescence analyses using an immune serum against a *bona fide* glycosomal protein, aldolase, showed no difference in the number of glycosomes per cell between the parental, Δ*fbpase* and Δ*fbpase*/FBPase cell lines (S2 Fig).

**Fig 9:**
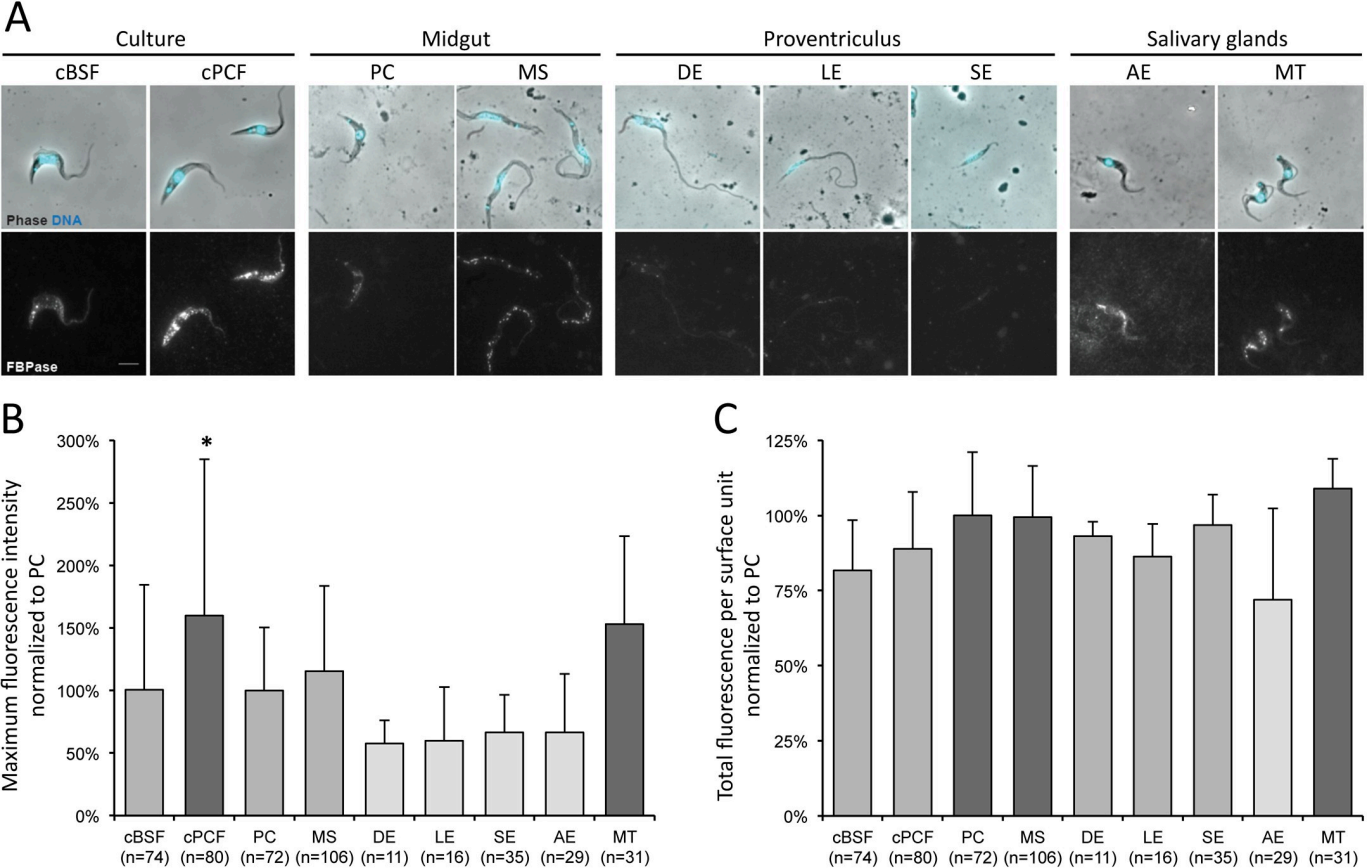
FBPase is expressed in all parasite cycle stages yet at variable levels. (A) Wild-type AnTat1.1E cultured bloodstream (cBSF) and procyclic (cPCF) forms, as well as parasites isolated from infected tsetse flies were fixed in methanol and stained with the anti-FBPase antibody (white) and DAPI (cyan). PC: procyclic trypomastigote, MS: mesocyclic trypomastigote, DE: dividing epimastigote, LE: long epimastigote, SE: short epimastigote, AE: attached epimastigote, and MT: metacyclic trypomastigote. (B-C) A region of interest was drawn on the phase picture of each individual cell and used for the quantification of the fluorescence provided by the anti-FBPase labelling. After background removal, the fluorescent signals were normalised (in %) to that obtained in PCF treated in the same experiment. The maximum fluorescence intensity (B) and total amount of fluorescence (C) corresponding to the FBPase expression levels were plotted according to the parasite stage. The total number of cells analysed from 3 independent experimental infections is indicated below the histogram bars for each stage. The different shades of grey represent groups of stages statistically identical between each other and different from those in the other groups according to a two-tailed ANOVA test with Tukey ad-hoc post-tests at 95% confidence for inter-group comparisons. * indicates the stage statistically different from all others with p<0.005.

In order to deepen the study of the *in vivo* role of FBPase, variations of the FBPase expression during the parasite cycle of wild-type trypanosomes were scrutinised by immunofluorescence analysis with an anti-FBPase antibody (Fig 9A). Assuming that the observed fluorescence is directly correlated to the amount of expressed proteins accessible to the antibody, all stages were seen to express FBPase in their glycosomes, yet in variable amounts (Fig 9A). The maximum signal intensities (Fig 9B) and the total amounts of fluorescent signal per surface unit (Fig 9C) were measured in a total of 454 individual cells isolated from more than 12 flies, further normalised to the values obtained on PCF cells from the same fly after removal of the background values, and finally plotted by stage in percentage of the fluorescent signals from PCF. The lowest maximum fluorescent signal intensities (Fig 9B) and total amounts of fluorescence per surface unit (Fig 9C) were observed in proventricular epimastigotes (DE, LE and SE) whereas the highest values were detected in salivary glands metacyclic forms (MT), and the differences between the two groups were statistically significant by ANOVA (p = 0.002, <0.001, <0.0001 and <0.0001 between MT Vs. DE, LE, SE and AE respectively by Tukey ad-hoc post-test on the maximum intensities, and p = 0.002 and <0.0001 between MT Vs. LE and AE for the total fluorescence per surface unit). The naturally lower FBPase expression in proventricular epimastigotes is in accordance with the absence of phenotype of the Δ*fbpase* null mutants in terms of morphotype switching ability (Fig 9A).

### FBPase is essential for production of metacyclic forms in proline rich-conditions

**Fig 10:**
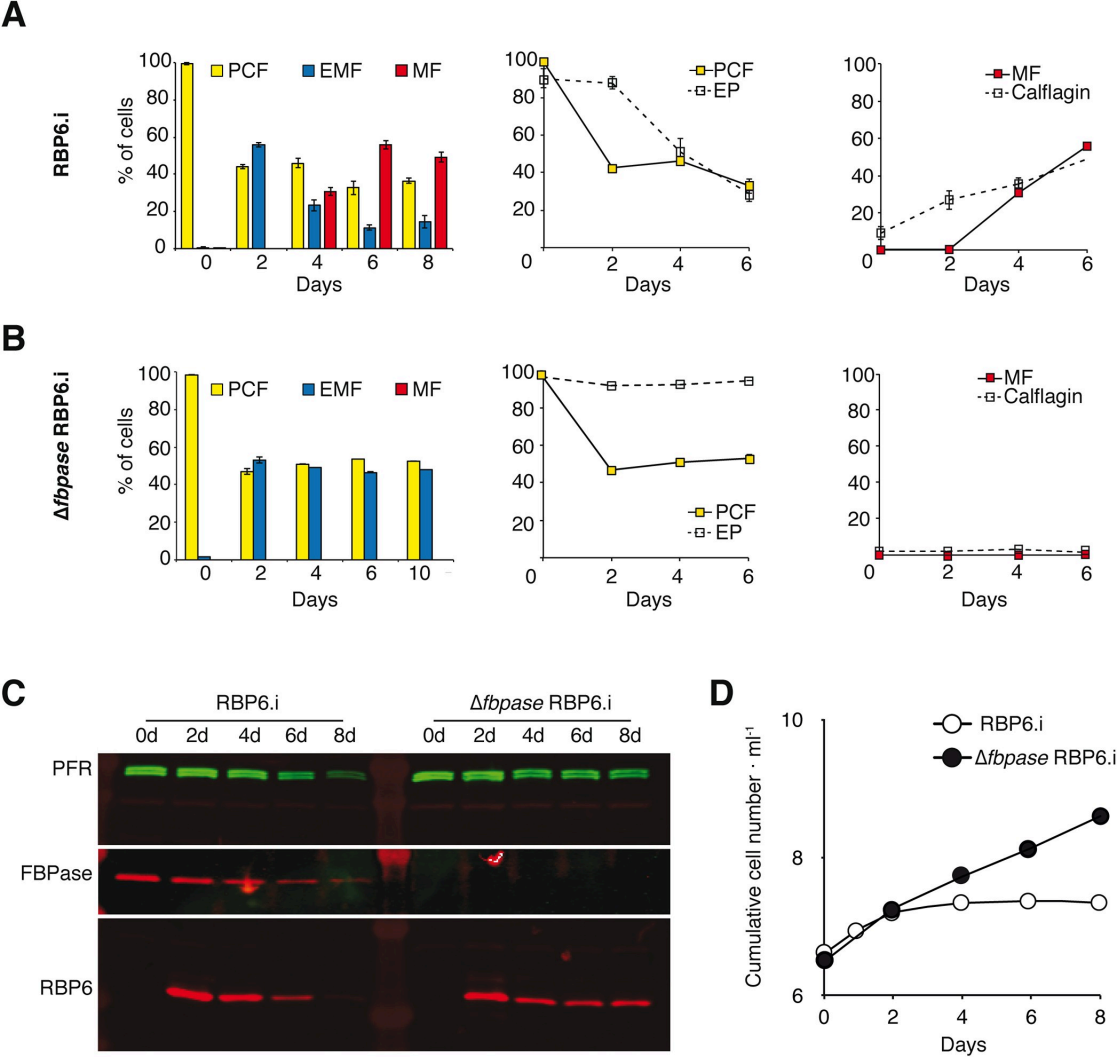
FBPase is essential for metacyclogenesis in culture. RBP6-induced differentiation kinetics is shown for the parental EATRO1125.T7T RBP6.i cell line (A) and the Δ*fbpase* RBP6.i mutant (B). RBP6 over-expression was induced at time 0 by addition of 1 μM tetracycline. Developmental stages were morphologically scored by cell size, shape and the relative position of the kinetoplast to the nucleus within the cell (n >100 cells per time point, biological triplicate, SEM). Expression of the stage-specific proteins EP and calflagin was quantified at the single cell level by flow cytometry and gated into two populations with low or high fluorescence. The left panels show histograms of morphotypes with PCF in yellow, EMF in blue and MF forms in red. The middle and right panels present morphotype fractions from the histogram (solid lines, data points colour matched to histogram) together with the fractions of stage marker expression levels (cells with high fluorescence/total number of cells, dashed lines) for EP and calflagin, respectively. (C) Western blot showing the RBP6 over-expression levels upon tetracycline induction of the indicated cell lines. PFR was used as loading control and the Δ*fbpase* knock out was verified by anti-FBPase. (D) Growth curves of the RBP6.i and Δ*fbpase* RBP6.i populations upon induction of RBP6 over-expression. C and D are from a representative replicate of the three experiments shown in A and B.

The fly passage phenotype observed may be due to an impairment of the parasite developmental program *per se* or to defects in migration, and/or to mobility defects during parasite migration in the tsetse and/or to non-adapted interactions with the tsetse environments. To distinguish between these possibilities, we tested the impact of the *FBPase* deletion in an *in vitro* model mimicking the parasite development upon inducible over-expression of the RNA binding protein 6 (RBP6), a central regulator of fly-stage differentiation [30]. The stage-specific morphology of induced cells, based on cell size and shape, as well as the relative position of the kinetoplast to the nucleus were monitored over the differentiation kinetics [31]. In PCF trypomastigotes the kinetoplast is at the posterior part of the cell, whereas in epimastigotes (EMF) the kinetoplast migrates to the opposite side of the nucleus and is found at the anterior part of the cell or in close proximity to the nucleus. Metacyclic trypomastigotes (MF) are smaller than EMF and PCF with the kinetoplast at the very end of the rounded posterior tip and a bloodstream form-like flagellar shape. In addition, we used two distinct stage-specific marker proteins for staging, *i*.*e*. EP procyclin (EP) for PCF [32] and calflagin for MF [33]. Calflagins are proteins localising to lipid rafts in the flagellar membrane and their amounts in bloodstream forms and MF are ten-fold enriched compared to that in PCF. Four days after the tetracycline-induction of RBP6 over-expression, ~30% of the cells (RBP6.i) had a characteristic MF morphology and after six days MF subpopulation reaches a peak at 55% (Fig 10A, left). Flow cytometry of RBP6.i cultures showed that EP expression was maintained in all cells during the first two days post induction and only then decreased proportionally to the fraction of the remaining PCF population (Fig 10A, middle). EP expression has previously been described in early salivary gland EMF [34]. The fraction of cells expressing calflagin constantly increased over time and attained the same percentage as the MF subpopulation after four and six days (Fig 10A, right). Previous reports showed that EP was not expressed in MF and that calflagins were detectable at basal levels in PCF but significantly up-regulated in MF [35, 36]. Additional evidence for developmental changes in these cultures is provided by RBP6 expression levels that decreased over time of the experiment (Fig 10C) and by a cell proliferation arrest (Fig 10D) in successfully differentiating cultures. The Δ*fbpase* RBP6.i mutant, in which RBP6 was conditionally over-expressed in the Δ*fbpase* background, was analysed in parallel under exactly comparable conditions (Fig 10B). Most importantly, the RBP6 expression levels as determined by quantitative western blotting (Fig 10C) were similar in the Δ*fbpase* and parental backgrounds over the entire time and did not decrease after 8 days. Induced Δ*fbpase* RBP6.i cells developed into EMF after two days but completely failed to differentiate into MF and consequently the fraction of EMF remained stable at around 50% for the duration of the experiment. EP and calflagin expression levels were not different between cell lines all over the time course and the population growth was maintained (Fig 10D). As the EP-positive population did not decrease, it is likely that the developmental blockage occurred in early EMF. Together, we can conclude that FBPase is absolutely essential for development in culture and hence this strong phenotype is cell autonomous.

## Discussion

The insect stages of *T*. *brucei* live in the midgut, the foregut and the salivary glands of the tsetse fly, which are considered glucose-free environment between the insect blood meals. In this context, trypanosomes need to produce G6P, the precursor of essential pathways, from non-glycolytic carbon sources. Production of hexose phosphates from proline through gluconeogenesis has recently been described for the PCF trypanosomes [21] and was consistent with the previously described metabolic switch toward proline when the parasite is incubated in glucose-depleted conditions [4, 37], as well as the requirement of an active proline metabolism to support development of trypanosomes in the insect midgut [3]. However, the pathway leading to F6P production as well as possible alternative gluconeogenic carbon sources used by the PCF were not addressed so far. Here, we show that two key steps of gluconeogenesis, *i*.*e*. production of PEP and production of F6P, are achieved by redundant reactions in PCF trypanosomes grown *in vitro*. However, the canonical FBPase gluconeogenic enzyme, is essential for the infection of the tsetse fly salivary glands where the differentiation of the attached epimastigote forms into free metacyclic infective forms occurs [35].

Production of the gluconeogenic precursor PEP from proline is performed by two different and complementary *T*. *brucei* glycosomal enzymes, *i*.*e*. PPDK and PEPCK. Indeed, abolition of G6P production from proline is only observed in the PPDK/PEPCK null background, but not in the Δ*ppdk* and Δ*pepck* single mutants. The implication of both the PEPCK and PPDK in gluconeogenesis has also been observed in *Leishmania mexicana*, however, the redundant effect was not investigated [20]. This redundancy between PPDK and PEPCK has also recently been observed in *Arabidopsis thaliana*, in which gluconeogenesis is critical to fuel the transition from seed to seedling [38]. To our knowledge, trypanosomatids and plants are the only eukaryotes known for using two distinct routes for the entry of organic acids into gluconeogenesis. In *L*. *mexicana*, PEPCK may participate in the entry of aspartate in promastigotes while PPDK is involved in the entry of alanine in amastigotes [20]. It is noteworthy, that this situation does not occur in *T*. *brucei*, since alanine and aspartate are not significantly consumed by the procyclic trypanosomes [4, 39, 40]. In *A*. *thaliana* PPDK participates in gluconeogenesis by remobilising amino acids such as alanine that give rise to pyruvate, while PEPCK is primarily involved in remobilisation of acetyl-CoA derived from lipids [38]. Interestingly, *T*. *brucei* is the only organism known so far to use PPDK in the gluconeogenic or in the glycolytic direction, depending on the carbon source availability. Indeed, we recently showed that in glucose-rich conditions PPDK plays a key role in the maintenance of the glycosomal ATP/ADP balance by converting PEP into pyruvate [12], while here we report that in the absence of glucose PPDK performs the reverse reaction to feed gluconeogenesis.

Important questions remain regarding the maintenance of the glycosomal ADP/ATP and redox balances in the PCF trypanosomes grown in glucose-free conditions. As mentioned above the glycosomal PPDK and PEPCK are critical for the maintenance of the glycosomal ADP/ATP balance by regenerating ATP when the parasite is fed with glucose [12]. However, in the absence of glucose, both enzymes work in the ATP-consuming direction with no glycosomal ATP-generating step in the gluconeogenic pathway. In this context, ATP could be provided to the enzymes involved in the gluconeogenetic pathway by another unknown glycosomal ADP/ATP-dependent enzyme, possibly encoded by one of the many hypothetical genes. Alternatively, glycosomal ATP could be regenerated through a carrier-mediated ADP/ATP exchange with the cytosolic compartment. Such a glycosomal carrier has not been described so far in trypanosomatids, although this hypothesis was previously proposed to explain the maintenance of the glycosomal ADP/ATP balance in the Δ*ppdk*/Δ*pepck* mutant grown in glucose-rich conditions [12]. It is important to note that this hypothetical glycosomal ADP/ATP exchange activity should be low enough to prevent the lethal turbo-explosion of the unusual glycolysis developed by trypanosomes [41]. Indeed, trypanosomes lack feedback regulation of the early steps of glycolysis, which is compatible with the glycosomal sequestration of the enzymes within glycosomes. However, partial relocation of the glycosomal enzymes within the cytosol induces accumulation of toxic glycolytic intermediates and ATP depletion by the so-called turbo-explosion. One may consider that a high capacity ATP/ADP exchanger located in the glycosomal membrane would lead to the same negative effect in the presence of glucose, unless the expression of this putative exchanger was controlled by glucose levels.

As expected, the *T*. *brucei* FBPase is involved in gluconeogenesis, as shown by a 6.5-fold reduction of F6P production from proline in the Δ*fbpase* mutant, which was restored by re-expressing an ectopic FBPase copy in the Δ*fbpase* background. However, to our surprise, the Δ*fbpase* and ^*RNAi*^FBPase.i mutants are viable in the glucose-free conditions, while blocking gluconeogenesis from both proline and glycerol in the Δ*ppdk*/Δ*pepck*/^*RNAi*^GK.i and Δ*ppdk*/Δ*pepck*/^*RNAi*^TIM.i cell lines compromises growth in the absence of glucose. These data, which are consistent with a residual production of ^13^C-enriched G6P from [U-^13^C]-proline in the Δ*fbpase* cell line, imply the existence of an alternative to FBPase for production of hexose phosphates from gluconeogenic carbon source(s). We must also take into account that the alternative to the FBPase reaction is probably not functional in *Leishmania major*, since FBPase has been shown to be essential for the development of the intracellular amastigote form as well as for the promastigote form growth in the absence of glucose [19]. Interestingly, the *T*. *brucei* gene potentially coding for SBPase is not present in the *Leishmania* genomes. The residual FBPase activity in the glycosomal fraction of the Δ*fbpase* cell line strongly supports the view that the product of this putative *T*. *brucei* SBPase is also involved in gluconeogenesis in the PCF trypanosomes. Our interpretation of the data is consistent with the previously characterisation in *Cyanobacteria* and in yeast of *FBPase*/*SBPase* genes showing a dual-function FBPase/SBPase [42–44].

Whatever this unknown alternative pathway is, it seems to be functional in the absence of FBPase in both *in vitro* cultured PCF and *in vivo* during the early cyclical development of midgut trypomastigote parasites. It is however not sufficient to allow Δ*fbpase* null mutants to colonise the tsetse fly vector salivary glands, demonstrating the crucial role of gluconeogenesis during this later part of the trypanosome cyclical development. These *in vivo* data showed that the rise of epimastigotes in the anterior midgut and proventriculus is not affected by the absence of FBPase. However, the absence of salivary gland infection suggests that either the proventricular epimastigotes have lost their capacity of maturation (e.g. to attached epimastigotes) and/or their abilty to migrate/orientate towards the salivary glands through the mouthparts and/or to attach to the salivary gland epithelium. The phenotype obtained *in vitro* in the RBP6-mediated differentiation model combined to the Δ*fbpase* null mutation is fully consistent with the fly infection data as EMF accumulate and no MF are detectable. Moreover, the development programme arrest in culture rules out that gluconeogenesis is mainly required to meet specific metabolic requirements of the fly alimentary tract micro-environments. A more defined staging of the developmental arrest is not possible *in vitro* as the EMF accumulating in culture are heterogenous in morphology and do not precisely match the natural morphologies present in the fly. It should also be remembered that the RBP6-induced differentiation is a useful tool yet a clearly “forced” model. Parasite development seems to depend on the expression of an alternative pathway or an enzymatic activity complementing the *FBPase* gene deletion. Once the identity of this bypass will be unravelled, we will have to test whether this phenotype may be due to a threshold effect of flux through the gluconeogenic pathway.

The growth of the PCF is compromised in glucose-free conditions only if triose phosphate production from proline and glycerol metabolism are together abolished in the Δ*ppdk*/Δ*pepck*/^*RNAi*^GK.i cell line, since the ^*RNAi*^GK.i and Δ*ppdk*/Δ*pepck* mutants are viable in these growth conditions. This implies that the glycerol conversion pathway is sufficient to feed gluconeogenesis. However, glycerol amounts are in the range of 5–10 μM in standard SDM79 medium (sera contain 50–100 μM glycerol [45, 46]) and should be consumed together with glucose by the PCF cells during preparation of the glucose-free medium. In the absence of glycerol in the medium, it could be produced from the medium compounds, such as phospholipids coming from fetal calf serum by the action of trypanosomal phospholipases [47] or from an endogenous carbon source. Interestingly, we previously reported the presence in PCF trypanosomes of a yet unknown endogenous carbon source detectable by excreted end products from its metabolism, acetate and succinate, in the absence of any extracellular carbon source [6, 23]. We are presently analysing this endogenous carbon source and its possible involvement in gluconeogenesis.

## Materials and methods

### Trypanosomes and cell cultures

The PCF of *T*. *brucei* EATRO1125.T7T (TetR-HYG T7RNAPOL-NEO) and AnTat1.1E [26] were cultivated at 27°C in the presence of 5% CO_2_ in SDM79 medium containing 10% (v/v) heat-inactivated fetal calf serum and 3.5 mg ml^-1^ hemin [48] or in a glucose-depleted medium derived from SDM79, called SDM79-GlcFree. This SDM79-GlcFree medium consists of a glucose-depleted SDM79 medium, containing 20% (v/v) heat-inactivated fetal calf serum, in which parental cells were cultured during 72 hours in order to consume the glucose coming from the serum and then diluted with the same volume of glucose-depleted SDM79 medium without serum to finally obtain SDM79-GlcFree. Glucose depletion was verified by NMR spectrometry analyses (with a detection threshold of glucose ≤ 1 μM) and to prevent import of residual glucose, a non-metabolised glucose analogue inhibiting glucose transport (*N*-acetyl-D-glucosamine, 50 mM) was added in the medium [21]. The same protocol was followed to produce SDM79-GlcFree and proline-depleted medium, except that the starting glucose-depleted SDM79 medium containing 100 μM proline, instead of 5 mM, was diluted with one volume of proline/glucose-depleted SDM79. Considering the rate of proline consumption of PCF trypanosomes, we estimated that the proline-depleted SDM79-GlcFree medium contains less than 20 μM proline. To estimate the cell density by the Alamer Blue assay, cells diluted in 200 μl of proline-depleted SDM79-GlcFree medium in which 1 μM to 100 mM proline was added by serial two-fold dilutions at a 2 x 10^6^ cell density, were incubated for 48 h at 27°C in microplates, before adding 20 μl of 0.49 mM Alamar Blue (Resazurin). Measurement of fluorescence was performed with the microplate reader Fluostar Optima (BMG Labtech) at 550 nm excitation wavelength and 600 nm emission wavelength.

### Gene knockout and rescue cell lines

The Δ*ppdk* mutant cell line was obtained by replacing both alleles of the *PPDK* gene (Tb927.11.3120, http://www.genedb.org/genedb/tryp/) by two different plasmids encoding the hygromycin resistance gene and the T7 RNA polymerase gene for the first one, and the neomycin resistance gene and the tetracycline repressor gene under the control of the T7 RNA polymerase promoter for the second one, as described before [49]. Replacement of the *PEPCK* gene (Tb927.2.4210) by the blasticidin (BSD) and puromycin (PAC) resistance markers via homologous recombination was described before (Δ*pepck* cell line) [22]. The Δ*ppdk*/Δ*pepck* double mutant was also described before [12]. Replacement of both alleles of the *FBPase* gene (Tb927.9.8720) by the blasticidin (BSD) and puromycin (PAC) resistance markers *via* homologous recombination was performed with DNA fragments containing a resistance marker gene flanked by the FBPase UTR sequences. Briefly, the pGEMt plasmid was used to clone an HpaI DNA fragment containing the BSD or PAC resistance marker gene preceded by the FBPase 5'-UTR fragment (729 bp) and followed by the FBPase 3'-UTR fragment (694 bp). The PAC resistance marker replaced one allele of the *FBPase* gene *via* homologous recombination and BSD resistance marker replaced the second allele. The EATRO1125.T7T parental cell line, which constitutively expresses the T7 RNA polymerase gene and the tetracycline repressor under the control of a T7 RNA polymerase promoter for tetracycline inducible expression (TetR-HYG T7RNAPOL-NEO) [50], was used to generate the FBPase knockout cell line. Transfection and selection of drug-resistant clones were performed as reported previously [11]. Transfected cells were selected in SDM79 medium containing hygromycin (25 μg ml^-1^), neomycin (10 μg ml^-1^), blasticidin (10 μg ml^-1^) and/or puromycin (1 μg ml^-1^). The selected cell line TetR-HYG T7RNAPOL-NEO Δ*fbpase*::BSD/Δ*fbpase*::PAC is called Δ*fbpase*. To produce the add-back Δ*fbpase*/FBPase rescue cell lines, a *FBPase* ectopic copy under control of the PARP promoter in the pLew100 expression vector, which contains the phleomycin resistance gene (kindly provided by E. Wirtz and G. Cross) [51], was introduced in the Δ*fbpase* null background. The pLew100-FBPase plasmid was generated by introduction the full length *FBPase* gene in the HindIII and BamHI restriction sites of the vector (Genecust). The Δ*fbpase*/FBPase cell line was selected in SDM79 medium containing phleomycin (5 μg ml^-1^) in addition to the 4 other antibiotics used to select the Δ*fbpase* cell line.

For experimental infections of Δtsetse flies, the Δ*fbpase* null mutant was generated in the pleomorphic AnTat1.1E PCF strain, using the same approach described above. Then, two distinct strategies were used to produce the add-back Δ*fbpase*/FBPase rescue cell lines in the AnTat1.1E Δ*fbpase* null background. The Δ*fbpase*/FBPase-1 cell line expresses a *FBPase* ectopic copy under control of the PARP promoter in the pLew100 expression vector as described above for the EATRO1125 Δ*fbpase*/FBPase cell line. The Δ*fbpase*/FBPase-2 cell line was generated by *in situ* re-insertion of one *FBPase* gene under the control of its endogenous UTRs. The 3.5 kb DNA fragment for *in situ* re-insertion consists on the *FBPase* coding sequence preceded by its 5' intergenic region (722 bp) and followed by its 3' intergenic region (662 bp), the phleomycin resistance gene (375 bp) and a second copy of the *FBPase* 3' intergenic region. The 3.5 kb DNA fragment flanked by two HpaI restriction sites was introduced into the pBluescript vector (Genecust) and the resulting plasmid was digested by HpaI before transfection of the AnTat1.1E Δ*fbpase* cell line. The Δ*fbpase*/FBPase-1 and Δ*fbpase*/FBPase-2 cell lines were selected in SDM79 medium containing blasticidin (10 μg ml^-1^), puromycin (1 μg ml^-1^) and phleomycin (5 μg ml^-1^).

### Inhibition of gene expression by RNAi

Accession numbers of genes targeted by RNAi are as follows; fructose-1,6-bisphosphatase (FBPase, Tb927.9.8720), glycerol kinase (GK, Tb927.9.12550-Tb927.9.12630), glycerol-3-phosphate dehydrogenase (GPDH, Tb927.8.3530) and triosephosphate isomerase (TIM, Tb927.11.5520). RNAi-mediated inhibition of gene expression was performed in the EATRO1125.T7T PCF by expression of stem-loop “sense-antisense” RNA molecules of the targeted sequences [50, 52] using the pLew100 expression vector [51].

To inhibit by RNAi the expression of the *FBPase* gene, a 597-bp fragment of the *FBPase* gene (from position 152 to 749) was introduced in the pLew100 vector to produce the pLew-FBPase-SAS plasmid. Briefly, a PCR-amplified 716-bp fragment, containing the antisense FBPase sequence with restriction sites added to the primers, was inserted into the HindIII and BamHI restriction sites of the pLew100 plasmid. The separate 615-bp PCR-amplified fragment containing the sense FBPase sequence was then inserted upstream of the antisense sequence, using HindIII and XhoI restriction sites (XhoI was introduced at the 3'-extremity of the antisense PCR fragment). The resulting plasmid pLew-FBPase-SAS contains a sense and antisense version of the targeted gene fragment, separated by a 89-bp fragment, under the control of a PARP promoter linked to a prokaryotic tetracycline operator. The same strategy was used to produce the pLew-GK-SAS, pLew-GPDH-SAS and pLew-TIM-SAS plasmids designed to inhibit the expression of the *GK*, *GPDH* and *TIM* genes, respectively. The size of the GK, GPDH and TIM targeted sequences are 617 bp (from position 460 to 1077), 622 bp (from position 218 to 840) and 558 bp (from position 67 to 625), respectively. The ^*RNAi*^FBPase mutant was generated by transfecting the EATRO1125.T7T parental cell line with the pLew-FBPase-SAS plasmid and selection in glucose-rich SDM79 medium containing hygromycin (25 μg ml^-1^), neomycin (10 μg ml^-1^) and phleomycin (5 μg ml^-1^). The triple mutant cell lines (Δ*ppdk/*Δ*pepck*/^*RNAi*^GK, Δ*ppdk/*Δ*pepck*/^*RNAi*^GPDH and Δ*ppdk/*Δ*pepck*/^*RNAi*^TIM) were obtained by introducing the relevant plasmid in the Δ*ppdk/*Δ*pepck* cell line, after selection in glucose-rich SDM79 medium containing hygromycin (25 μg ml^-1^), neomycin (10 μg ml^-1^), phleomycin (5 μg ml^-1^), blasticidin (10 μg ml^-1^) and puromycin (1 μg ml^-1^). Aliquots were frozen in liquid nitrogen to provide stocks of each line that had not been cultivated long term in medium. Induction of RNAi cell lines was performed by addition of 1 μg ml^-1^ tetracycline.

### Preparation of glycosomal fractions and enzymatic activities

Subcellular fraction enriched in glycosomes was prepared by differential centrifugation as described in [53], after homogenising the cells with silicon carbide as grinding material. Briefly, 2 x 10^9^ cells were washed once in 10 ml of STE (25 mM Tris, 1 mM EDTA, 250 mM sucrose pH 7.8). After centrifugation, the pellet was resuspended in 0.15 ml of homogenisation buffer STE (STE supplemented with ‘Complete EDTA-Free’ protease-inhibitor cocktail, Roche Applied Science, Mannheim, Germany) and ground in a pre-chilled mortar with 1.5 gr of wet-weight silicon carbide per gr of cell pellet. The cells were microscopically checked for at least 90% disruption. The cell lysate was diluted in 7 ml of homogenisation buffer, centrifuged at 1,000 g and then at 5,000 g for 10 min each, at 4°C. The supernatant was centrifuged at 33,000 g for 10 min at 4°C to yield the cytosolic fraction and the glycosome-enriched pellet, which was washed once with 1 ml of STE, centrifuged at 33,000 g for 10 min at 4°C before resuspension in 0.1 ml of STE.

The FBPase activity of aliquots of the glycosomal fractions was measured following the reduction of NADP^+^ at 340 nm in the presence of 100 mM triethanolamine (pH 7.6), 2 mM MgCl_2_, 0.1 mM EDTA, 0.3 mM NADP^+^, 10 mM F1,6BP, 25 μg ml^-1^ glucose-6-phosphate isomerase and 25 μg ml^-1^ glucose-6-phosphate dehydrogenase. The glycerol kinase activity was measured at 340 nm *via* oxidation of NADH according to published procedures [54].

### Western blot analyses

Total protein extracts (5 x 10^6^ cells), or glycosomal and cytosolic fractions, of parental (AnTat1.1E, EATRO1125.T7T) or mutant PCF of *T*. *brucei* were separated by SDS-PAGE (10%) and immunoblotted on TransBlot Turbo Midi-size PVFD Membranes (Bio-Rad) [55]. Immunodetection was performed as described [55, 56] using as primary antibodies, the rabbit anti-FBPase (1:1,000, gift from P. Michels, Edinburgh, UK), the rabbit anti-SBPase (1:250, gift from P. Michels, Edinburgh, UK), the rabbit anti-GK (1:5,000, gift from P. Michels, Edinburgh, UK), the rabbit anti-G6PDH (1:1,000, gift from P. Michels, Edinburgh, UK), the rabbit anti-TIM (1:1,000, gift from P. Michels, Edinburgh, UK), the rabbit anti-PFR (1:10,000), the rabbit anti-enolase (1:100,000, gift from P. Michels, Edinburgh, UK), the rat anti-PEPCK (1:1,000, gift from T. Seebeck, Bern, Switzerland) [22], the rabbit anti-GPDH (1:1,000) [57], the rabbit anti-hsp60 (1:10,000) [58], the rabbit anti-PPDK (1:1,000) [11], anti-EP mouse monoclonal TBRP1/247 (1:500) (Biozol), anti-calflagin (1:1,000, gift of D. Engman, Chicago, USA [59]) and the rabbit anti-RBP6 (1:1,000, gift from C. Tschudi, New Haven, USA) [30]. Anti-rabbit or anti-rat IgG conjugated to horseradish peroxidase (Bio-Rad, 1:5000 dilution) was used as secondary antibody. Revelation was performed using the Clarity western ECL Substrate as described by the manufacturer (Bio-Rad). Images were acquired and analysed with the ImageQuant LAS 4000 luminescent image analyser.

### *In vitro* differentiation of PCF expressing RBP6

The *RBP6* gene was amplified by PCR and cloned via HindIII/BamHI into the pLew100v5b1d vector (pLew100v5 modified with a blasticidin resistance gene *BSD*). The Δ*fbpase* cell and its parental EATRO1125.T7T cell line were transfected with the pLew100v5-RBP6 linearised with NotI in pools to generate the Δ*fbpase* RBP6 cell line with the genotype *TETR*::*HYG T7RNAP*::*NEO* Δ*fbpase*::*BLAS/*Δ*fbpase*::*PAC RBP6*^*Ti*^::*BLE* and RBP6, respectively. *In vitro* differentiation experiments were done as described in [30] in SDM79 medium without glucose in the presence of 50 mM *N*-acetyl-D-glucosamine and 10% (v/v) heat-inactivated fetal calf serum. The faster kinetics of differentiation observed in our experiments as compared to that described in [30] is likely due to the different parental backgrounds (Lister 427 29:13 *versus* EATRO1125.T7T) or to the different media (Cunningham’s medium [60] *versus* modified SDM79) that were used in the two labs.

### Flow cytometry analysis of stage-specific surface marker

2 × 10^7^ cells were harvested and fixed overnight with 2% paraformaldehyde in PBS at 4°C, washed thrice with PBS and resuspended in 500 μl PBS. Cells were incubated with monoclonal mouse anti-procyclin EP (TBRP1/247, 1:500) in 1% BSA. For calflagin detection 3 × 10^7^ cells were permeabilised with 0.2% NP-40 for 5 min prior incubation with polyclonal mouse α-calflagin (1:1,000) [59] in 1% BSA. Alexa Fluor 488-conjugated goat antibodies were used as secondary antibodies. Samples were analysed with a FACSCalibur cell analyser (Becton Dickinson) and data were evaluated with the FlowJo 8.8.6 software.

### Mass spectrometry analyses of [^13^C]-incorporation into cellular metabolites

EATRO1125.T7T parental and mutant cell lines grown in SDM79 medium were washed twice with PBS and resuspended in PBS containing [U-^13^C]-proline (or [U-^13^C]-glycerol) with or without the same amount of non-labelled glucose. The cells were incubated for 2 h at 27°C before being collected on filters by fast filtration preparation (2 x 10^7^ cells per filter) for mass spectrometry analysis, as described before [22]. Metabolites were analysed by ion-exchange chromatography coupled with tandem mass spectrometry (IC-MS/MS) using the method described by Bolten *et al*. [61]. Retention time on the column and multiple reactions monitoring (MRM) transition of each analysed metabolite are shown in Table 1 of [21]. The ^13^C mass isotopomer distribution of intracellular metabolites was determined from relevant isotopic clusters in the IC-MS/MS analysis, according to Kiefer *et al*. [62]. ^13^C mass isotopomer distribution measurements were performed using a triple quadrupole mass spectrometer (4000Qtrap, Applied Biosystems). To obtain [^13^C]-labeling patterns (^13^C isotopologues), isotopic clusters were corrected for the natural abundance of isotopes other than ^13^C, using the in-house software IsoCor (available at MetaSys) [63].

### Analysis of excreted end products from glucose and glycerol metabolism by proton NMR

1 x 10^8^ *T*. *brucei* PCF were collected by centrifugation at 1,400 g for 10 min, washed once with phosphate-buffered saline (PBS) and incubated in 5 ml of PBS supplemented with 2 g l^-1^ NaHCO_3_ (pH 7.4). Cells were maintained for 6 h at 27°C in incubation buffer containing 20 μmol of D-glucose or [U-^13^C]-glycerol (4 mM). The integrity of the cells during the incubation was checked by microscopic observation. The supernatant was collected and 50 μl of maleate solution in D_2_O (10 mM) was added as internal reference. H-NMR spectra were performed at 500.19 MHz on a Bruker Avance III 500 HD spectrometer equipped with a 5 mm cryoprobe Prodigy. Measurements were recorded at 25°. Acquisition conditions were as follows: 90° flip angle, 5,000 Hz spectral width, 32 K memory size, and 9.3 sec total recycle time. Measurements were performed with 64 scans for a total time close to 10 min 30 sec. Resonances of the obtained spectra were integrated and metabolites concentrations were calculated using the ERETIC2 NMR quantification Bruker program.

### Tsetse fly maintenance, infection and dissection

Tsetse flies (*Glossina morsitans morsitans*) were maintained, infected and dissected at the Institut Pasteur as previously described [28]. Teneral males were collected 24 h to 48 h post-eclosion and artificially fed through a silicone membrane with 6–9 x 10^6^ parasites ml^-1^ in SDM79 medium supplemented with 10% FCS for their first meal. Flies were then maintained in Roubaud cages for one month at 26°C and 60% hygrometry and fed twice a week with mechanically defibrinated sheep blood. Flies were starved for at least 48 h before being individually dissected 28 days after ingestion of the infected meal. Salivary glands were first rapidly dissected into a drop of PBS. The whole tsetse alimentary tract was then dissected and arranged lengthways for assessment of parasite presence. The proventriculus and anterior midgut were physically separated from the posterior midgut in a distinct PBS drop. Tissues were dilacerated to allow parasites to spread in PBS, parasites were recovered and treated for further experiments no more than 15 min after dissection.

### Immunofluorescence analysis

For immunofluorescence, parasites were rapidly allowed to settle on poly-lysine coated slides until drying. Cells were fixed for 10 sec in methanol at -20°C and re-hydrated in PBS during 10 min. Slides were then incubated for 45 min at 37°C with the anti-FBPase primary antibody diluted at 1:10 in PBS with 0,1% bovine serum albumin. Slides were washed in PBS and incubated for 30 min at 37°C with the appropriate anti-rabbit secondary antibody coupled to an Alexa 488 fluorophore (Invitrogen). Slides were then stained with DAPI for visualisation of their kinetoplast and nuclear DNA contents and mounted under coverslip with Prolong antifade reagent (Invitrogen). IFA experiments were repeated on trypanosomes issued from 2 to 4 flies and from 6 distinct experimental infections. Slides were finally observed with a DMI4000 epifluorescence microscope (Leica) and images were captured with a Horca 03G5 camera (Hamamastu).

### Measurements and analyses

Normalisation of signals was carried out by parallel manipulation of min/max signals in ImageJ (NIH). Statistical analyses and plots were performed with XLSTAT 2015.4.01 (Addinsoft) and Excel 2011 (Microsoft), respectively. Statistical analyses include ANOVA with Tukey ad-hoc post-tests for inter-group comparison with 95% confidence.

## Supporting information

S1 Fig IC-MS/MS analysis of intracellular metabolites after isotopic labelling with [U-^13^C]-labelled carbon sources. (PDF)

S2 Fig The number of glycosomes is not affected by the *FBPase* gene knockout. (PDF)

S1 Table Effect of glucose deprivation on metabolite pools. (XLSX)

## References

[1] Buscher P, Cecchi G, Jamonneau V, Priotto G. Human African trypanosomiasis. Lancet. 2017;390:2397–2409. 10.1016/S0140-6736(17)31510-6 . 28673422

[2] Bringaud F, Barrett MP, Zilberstein D. Multiple roles of proline transport and metabolism in trypanosomatids. Frontiers in Bioscience 2012;17:349–374. 10.2741/3931 22201748

[3] Mantilla BS, Marchese L, Casas-Sanchez A, Dyer NA, Ejeh N, Biran M, et al Proline metabolism is essential for *Trypanosoma brucei brucei* survival in the tsetse vector. PLoS Pathog. 2017;13:e1006158 10.1371/journal.ppat.1006158 28114403 PMC5289646

[4] Coustou V, Biran M, Breton M, Guegan F, Riviere L, Plazolles N, et al Glucose-induced remodeling of intermediary and energy metabolism in procyclic *Trypanosoma brucei*. J Biol Chem. 2008;283:16342–16354. 10.1074/jbc.M709592200 18430732

[5] Bringaud F, Riviere L, Coustou V. Energy metabolism of trypanosomatids: adaptation to available carbon sources. Mol Biochem Parasitol. 2006;149:1–9. 10.1016/j.molbiopara.2006.03.017 16682088

[6] Bringaud F, Biran M, Millerioux Y, Wargnies M, Allmann S, Mazet M. Combining reverse genetics and NMR-based metabolomics unravels trypanosome-specific metabolic pathways. Mol Microbiol. 2015;96:917–926. 10.1111/mmi.12990 25753950

[7] Opperdoes FR, Borst P. Localization of nine glycolytic enzymes in a microbody-like organelle in *Trypanosoma brucei*: the glycosome. FEBS Lett. 1977;80:360–364. doi: 0014-5793(77)80476-6. 142663 10.1016/0014-5793(77)80476-6

[8] Gualdron-Lopez M, Brennand A, Hannaert V, Quinones W, Caceres AJ, Bringaud F, et al When, how and why glycolysis became compartmentalised in the Kinetoplastea. A new look at an ancient organelle. Int J Parasitol. 2012;42:1–20. 10.1016/j.ijpara.2011.10.007 22142562

[9] Besteiro S, Biran M, Biteau N, Coustou V, Baltz T, Canioni P, et al Succinate secreted by *Trypanosoma brucei* is produced by a novel and unique glycosomal enzyme, NADH-dependent fumarate reductase. J Biol Chem. 2002;277:38001–38012. 10.1074/jbc.M201759200 12138089

[10] Coustou V, Besteiro S, Riviere L, Biran M, Biteau N, Franconi JM, et al A mitochondrial NADH-dependent fumarate reductase involved in the production of succinate excreted by procyclic *Trypanosoma brucei*. J Biol Chem. 2005;280:16559–16570. 10.1074/jbc.M500343200 15718239

[11] Bringaud F, Baltz D, Baltz T. Functional and molecular characterization of a glycosomal PPi-dependent enzyme in trypanosomatids: pyruvate, phosphate dikinase. Proc Natl Acad Sci USA. 1998;95:7963–7968. 9653123 10.1073/pnas.95.14.7963 PMC20912

[12] Deramchia K, Morand P, Biran M, Millerioux Y, Mazet M, Wargnies M, et al Contribution of pyruvate phosphate dikinase in the maintenance of the glycosomal ATP/ADP balance in the *Trypanosoma brucei* procyclic form. J Biol Chem. 2014;289:17365–17378. 10.1074/jbc.M114.567230 24794874 PMC4067170

[13] Van Hellemond JJ, Opperdoes FR, Tielens AG. Trypanosomatidae produce acetate via a mitochondrial acetate:succinate CoA transferase. Proc Natl Acad Sci USA. 1998;95:3036–3041. 9501211 10.1073/pnas.95.6.3036 PMC19690

[14] Riviere L, van Weelden SW, Glass P, Vegh P, Coustou V, Biran M, et al Acetyl:succinate CoA-transferase in procyclic *Trypanosoma brucei*. Gene identification and role in carbohydrate metabolism. J Biol Chem. 2004;279:45337–45346. 10.1074/jbc.M407513200 15326192

[15] Millerioux Y, Morand P, Biran M, Mazet M, Moreau P, Wargnies M, et al ATP synthesis-coupled and -uncoupled acetate production from acetyl-CoA by the mitochondrial acetate:succinate CoA-transferase and acetyl-CoA thioesterase in *Trypanosoma*. J Biol Chem. 2012;287:17186–17197. 10.1074/jbc.M112.355404 22474284 PMC3366814

[16] Van Weelden SW, Fast B, Vogt A, Van Der Meer P, Saas J, Van Hellemond JJ, et al Procyclic *Trypanosoma brucei* do not use Krebs cycle activity for energy generation. J Biol Chem. 2003;278:12854–12863. 10.1074/jbc.M213190200 12562769

[17] Stokes MJ, Guther ML, Turnock DC, Prescott AR, Martin KL, Alphey MS, et al The synthesis of UDP-N-acetylglucosamine is essential for bloodstream form *Trypanosoma brucei in vitro* and *in vivo* and UDP-N-acetylglucosamine starvation reveals a hierarchy in parasite protein glycosylation. J Biol Chem. 2008;283:16147–16161. 10.1074/jbc.M709581200 18381290 PMC2414269

[18] Kovarova J, Barrett MP. The Pentose Phosphate Pathway in Parasitic Trypanosomatids. Trends Parasitol. 2016;32:622–634. 10.1016/j.pt.2016.04.010 27174163

[19] Naderer T, Ellis MA, Sernee MF, De Souza DP, Curtis J, Handman E, et al Virulence of *Leishmania major* in macrophages and mice requires the gluconeogenic enzyme fructose-1,6-bisphosphatase. Proc Natl Acad Sci USA. 2006;103:5502–5507. 10.1073/pnas.0509196103 16569701 PMC1459384

[20] Rodriguez-Contreras D, Hamilton N. Gluconeogenesis in *Leishmania mexicana*: contribution of glycerol kinase, phosphoenolpyruvate carboxykinase, and pyruvate phosphate dikinase. J Biol Chem. 2014;289:32989–33000. 10.1074/jbc.M114.569434 25288791 PMC4239644

[21] Allmann S, Morand P, Ebikeme C, Gales L, Biran M, Hubert J, et al Cytosolic NADPH homeostasis in glucose-starved procyclic *Trypanosoma brucei* relies on malic enzyme and the pentose phosphate pathway fed by gluconeogenic flux. J Biol Chem. 2013;288:18494–18505. 10.1074/jbc.M113.462978 23665470 PMC3689991

[22] Ebikeme C, Hubert J, Biran M, Gouspillou G, Morand P, Plazolles N, et al Ablation of succinate production from glucose metabolism in the procyclic trypanosomes induces metabolic switches to the glycerol 3-phosphate/dihydroxyacetone phosphate shuttle and to proline metabolism. J Biol Chem. 2010;285:32312–32324. 10.1074/jbc.M110.124917 20702405 PMC2952232

[23] Millerioux Y, Ebikeme C, Biran M, Morand P, Bouyssou G, Vincent IM, et al The threonine degradation pathway of the *Trypanosoma brucei* procyclic form: the main carbon source for lipid biosynthesis is under metabolic control. Mol Microbiol. 2013;90:114–129. 10.1111/mmi.12351 23899193 PMC4034587

[24] Guther ML, Urbaniak MD, Tavendale A, Prescott A, Ferguson MA. High-confidence glycosome proteome for procyclic form *Trypanosoma brucei* by epitope-tag organelle enrichment and SILAC proteomics. J Proteome Res. 2014;13:2796–2806. 10.1021/pr401209w 24792668 PMC4052807

[25] Gualdron-Lopez M, Chevalier N, Van Der Smissen P, Courtoy PJ, Rigden DJ, Michels PAM. Ubiquitination of the glycosomal matrix protein receptor PEX5 in *Trypanosoma brucei* by PEX4 displays novel features. Biochim Biophys Acta. 2013;1833:3076–3092. 10.1016/j.bbamcr.2013.08.008 23994617

[26] Le Ray D, Barry JD, Easton C, K. V. First tsetse fly transmission of the "AnTat" serodeme of *Trypanosoma brucei*. Ann Soc Belg Med Trop. 1977;57:369–381. 610616

[27] Millerioux Y, Mazet M, Bouyssou G, Allmann S, Kiema T-R, Bertiaux E, et al *De novo* biosynthesis of sterols and fatty acids in the *Trypanosoma brucei* procyclic form: carbon source preferences and metabolic flux redistributions. PLoS Pathog. 2018;14:e1007116 10.1371/journal.ppat.1007116 29813135 PMC5993337

[28] Rotureau B, Subota I, Bastin P. Molecular bases of cytoskeleton plasticity during the *Trypanosoma brucei* parasite cycle. Cell Microbiol. 2011;13:705–716. 10.1111/j.1462-5822.2010.01566.x 21159115

[29] Pradel LC, Bonhivers M, Landrein N, Robinson DR. NIMA-related kinase TbNRKC is involved in basal body separation in *Trypanosoma brucei*. J Cell Sci. 2006;119:1852–1863. 10.1242/jcs.02900 16608878

[30] Kolev NG, Ramey-Butler K, Cross GA, Ullu E, Tschudi C. Developmental progression to infectivity in *Trypanosoma brucei* triggered by an RNA-binding protein. Science. 2012;338:1352–1353. 10.1126/science.1229641 23224556 PMC3664091

[31] Rotureau B, Van Den Abbeele J. Through the dark continent: African trypanosome development in the tsetse fly. Front Cell Infect Microbiol. 2013;3:53 10.3389/fcimb.2013.00053 24066283 PMC3776139

[32] Vassella E, Acosta-Serrano A, Studer E, Lee SH, Englund PT, Roditi I. Multiple procyclin isoforms are expressed differentially during the development of insect forms of *Trypanosoma brucei*. J Mol Biol. 2001;312:597–607. 10.1006/jmbi.2001.5004 11575917

[33] Emmer BT, Daniels MD, Taylor JM, Epting CL, Engman DM. Calflagin inhibition prolongs host survival and suppresses parasitemia in *Trypanosoma brucei* infection. Eukaryot Cell. 2010;9:934–942. 10.1128/EC.00086-10 20418379 PMC2901653

[34] Richardson JP, Jenni L, Beecroft RP, Pearson TW. Procyclic tsetse fly midgut forms and culture forms of African trypanosomes share stage- and species-specific surface antigens identified by monoclonal antibodies. J Immunol. 1986;136:2259–2264. 3512712

[35] Rotureau B, Subota I, Buisson J, Bastin P. A new asymmetric division contributes to the continuous production of infective trypanosomes in the tsetse fly. Development. 2012;139:1842–1850. 10.1242/dev.072611 22491946

[36] Christiano R, Kolev NG, Shi H, Ullu E, Walther TC, Tschudi C. The proteome and transcriptome of the infectious metacyclic form of *Trypanosoma brucei* define quiescent cells primed for mammalian invasion. Mol Microbiol. 2017;106:74–92. 10.1111/mmi.13754 . 28742275 PMC5607103

[37] Lamour N, Riviere L, Coustou V, Coombs GH, Barrett MP, Bringaud F. Proline metabolism in procyclic *Trypanosoma brucei* is down-regulated in the presence of glucose. J Biol Chem. 2005;280:11902–11910. 10.1074/jbc.M414274200 15665328

[38] Eastmond PJ, Astley HM, Parsley K, Aubry S, Williams BP, Menard GN, et al *Arabidopsis* uses two gluconeogenic gateways for organic acids to fuel seedling establishment. Nat Commun. 2015;6:6659 10.1038/ncomms7659 25858700 PMC4403315

[39] Spitznagel D, Ebikeme C, Biran M, NAB N., Bringaud F, Henehan GT, et al Alanine aminotransferase of *Trypanosoma brucei*—a key role in proline metabolism in procyclic life forms. FEBS J. 2009;276:7187–7199. 10.1111/j.1742-4658.2009.07432.x 19895576

[40] Ong HB, Lee WS, Patterson S, Wyllie S, Fairlamb AH. Homoserine and quorum-sensing acyl homoserine lactones as alternative sources of threonine: a potential role for homoserine kinase in insect-stage *Trypanosoma brucei*. Mol Microbiol. 2014;95:143–156. 10.1111/mmi.12853 25367138 PMC4460637

[41] Haanstra JR, van Tuijl A, Kessler P, Reijnders W, Michels PA, Westerhoff HV, et al Compartmentation prevents a lethal turbo-explosion of glycolysis in trypanosomes. Proc Natl Acad Sci USA. 2008;105:17718–17723. 10.1073/pnas.0806664105 19008351 PMC2584722

[42] Kuznetsova E, Xu L, Singer A, Brown G, Dong A, Flick R, et al Structure and activity of the metal-independent fructose-1,6-bisphosphatase YK23 from *Saccharomyces cerevisiae*. J Biol Chem. 2010;285:21049–21059. 10.1074/jbc.M110.118315 20427268 PMC2898295

[43] Ganapathy U, Marrero J, Calhoun S, Eoh H, de Carvalho LP, Rhee K, et al Two enzymes with redundant fructose bisphosphatase activity sustain gluconeogenesis and virulence in *Mycobacterium tuberculosis*. Nat Commun. 2015;6:7912 10.1038/ncomms8912 26258286 PMC4535450

[44] Feng L, Sun Y, Deng H, Li D, Wan J, Wang X, et al Structural and biochemical characterization of fructose-1,6/sedoheptulose-1,7-bisphosphatase from the *Cyanobacterium synechocystis* strain 6803. FEBS J. 2014;281:916–926. 10.1111/febs.12657 24286336

[45] Jansson PA, Smith U, Lonnroth P. Interstitial glycerol concentration measured by microdialysis in two subcutaneous regions in humans. Am J Physiol. 1990;258:E918–922. 10.1152/ajpendo.1990.258.6.E918 2193533

[46] Samra JS, Ravell CL, Giles SL, Arner P, Frayn KN. Interstitial glycerol concentration in human skeletal muscle and adipose tissue is close to the concentration in blood. Clin Sci (Lond). 1996;90:453–456. 8697714 10.1042/cs0900453

[47] Belaunzaran ML, Lammel EM, de Isola EL. Phospholipases a in trypanosomatids. Enzyme Res. 2011;2011:392082 10.4061/2011/392082 21603263 PMC3092542

[48] Brun R, Schonenberger M. Cultivation and in vitro cloning or procyclic culture forms of *Trypanosoma brucei* in a semi-defined medium. Acta Trop. 1979;36:289–292. 43092

[49] Coustou V, Besteiro S, Biran M, Diolez P, Bouchaud V, Voisin P, et al ATP generation in the *Trypanosoma brucei* procyclic form: Cytosolic substrate level phosphorylation is essential, but not oxidative phosphorylation. J Biol Chem. 2003;278:49625–49635. 10.1074/jbc.M307872200 . 14506274

[50] Bringaud F, Robinson DR, Barradeau S, Biteau N, Baltz D, Baltz T. Characterization and disruption of a new *Trypanosoma brucei* repetitive flagellum protein, using double-stranded RNA inhibition. Mol Biochem Parasitol. 2000;111:283–297. 11163437 10.1016/s0166-6851(00)00319-4

[51] Wirtz E, Leal S, Ochatt C, Cross GA. A tightly regulated inducible expression system for conditional gene knock-outs and dominant-negative genetics in *Trypanosoma brucei*. Mol Biochem Parasitol. 1999;99:89–101. 10215027 10.1016/s0166-6851(99)00002-x

[52] Ngo H, Tschudi C, Gull K, Ullu E. Double-stranded RNA induces mRNA degradation in *Trypanosoma brucei*. Proc Natl Acad Sci USA. 1998;95:14687–14692. 9843950 10.1073/pnas.95.25.14687 PMC24510

[53] Opperdoes FR, Borst P, Spits H. Particle-bound enzymes in the bloodstream form of *Trypanosoma brucei*. Eur J Biochem. 1977;76:21–28. 195809 10.1111/j.1432-1033.1977.tb11566.x

[54] Kralova I, Rigden DJ, Opperdoes FR, Michels PA. Glycerol kinase of *Trypanosoma brucei*. Cloning, molecular characterization and mutagenesis. Eur J Biochem. 2000;267:2323–2333. 10759857 10.1046/j.1432-1327.2000.01238.x

[55] Harlow E, Lane D, editors. Antibodies: a laboratory manual: Cold Spring Harbor Laboratory Press; 1988.

[56] Sambrook J, Fritsch EF, Maniatis T, editors. Molecular cloning: a laboratory manual. 2 ed New York: Cold Spring Harbor Laboratory Press; 1989.

[57] Denise H, Giroud C, Barrett MP, Baltz T. Affinity chromatography using trypanocidal arsenical drugs identifies a specific interaction between glycerol-3-phosphate dehydrogenase from *Trypanosoma brucei* and Cymelarsan. Eur J Biochem. 1999;259:339–346. 9914512 10.1046/j.1432-1327.1999.00048.x

[58] Bringaud F, Peyruchaud S, Baltz D, Giroud C, Simpson L, Baltz T. Molecular characterization of the mitochondrial heat shock protein 60 gene from *Trypanosoma brucei*. Mol Biochem Parasitol. 1995;74:119–123. 8719252 10.1016/0166-6851(95)02486-7

[59] Tyler KM, Fridberg A, Toriello KM, Olson CL, Cieslak JA, Hazlett TL, et al Flagellar membrane localization via association with lipid rafts. J Cell Sci. 2009;122:859–866. 10.1242/jcs.037721 19240119 PMC2714428

[60] Cunningham I. New culture medium for maintenance of tsetse tissues and growth of trypanosomatids. J Protozool. 1977;24:325–329. PubMed 881656 10.1111/j.1550-7408.1977.tb00987.x

[61] Bolten CJ, Kiefer P, Letisse F, Portais JC, Wittmann C. Towards appropriate sampling for metabolome analysis of microorganisms. Anal chem. 2007;79:3843–3849. 10.1021/ac0623888 17411014

[62] Kiefer P, Nicolas C, Letisse F, Portais JC. Determination of carbon labeling distribution of intracellular metabolites from single fragment ions by ion chromatography tandem mass spectrometry. Anal Biochem. 2007;360:182–188. 10.1016/j.ab.2006.06.032 17134674

[63] Millard P, Letisse F, Sokol S, Portais JC. IsoCor: correcting MS data in isotope labeling experiments. Bioinformatics. 2012;28:1294–1296. 10.1093/bioinformatics/bts127 22419781

