# Supplementary material for: Gluconeogenesis is essential for trypanosome development in the tsetse fly vector
Source: PLoS Pathog. 2018 Dec 17;14(12):e1007502. doi: 10.1371/journal.ppat.1007502 (PMC6312356; doi:10.1371/journal.ppat.1007502)

S1 Fig. IC-MS/MS analysis of intracellular metabolites after isotopic labelling with [U-^{13}C]-labelled carbon sources. The EATRO1125.T7T parental cell line was incubated for 2 h in PBS containing 2 mM [U-^{13}C]- proline, 2 mM [U-^{13}C]-glycerol or 2 mM [U-^{13}C]-glucose as indicated. The figure shows enrichment of key glycolytic intermediates at 0 to 6 carbon positions (m0 to m6, colour code indicated below) with ^{13}C expressed as percentage of all corresponding molecules (MID; Mass Isotopomer Distribution). Abbreviations: G6P, glucose 6-phosphate; F6P, fructose 6-phosphate; Gly3P, glycerol 3-phosphate; M6P, mannose 6-phosphate; 2/3PG, 2- or 3-phosphoglycerate (these two metabolites are undistinguished by IC-MS/MS); PEP, phosphoenolpyruvate.

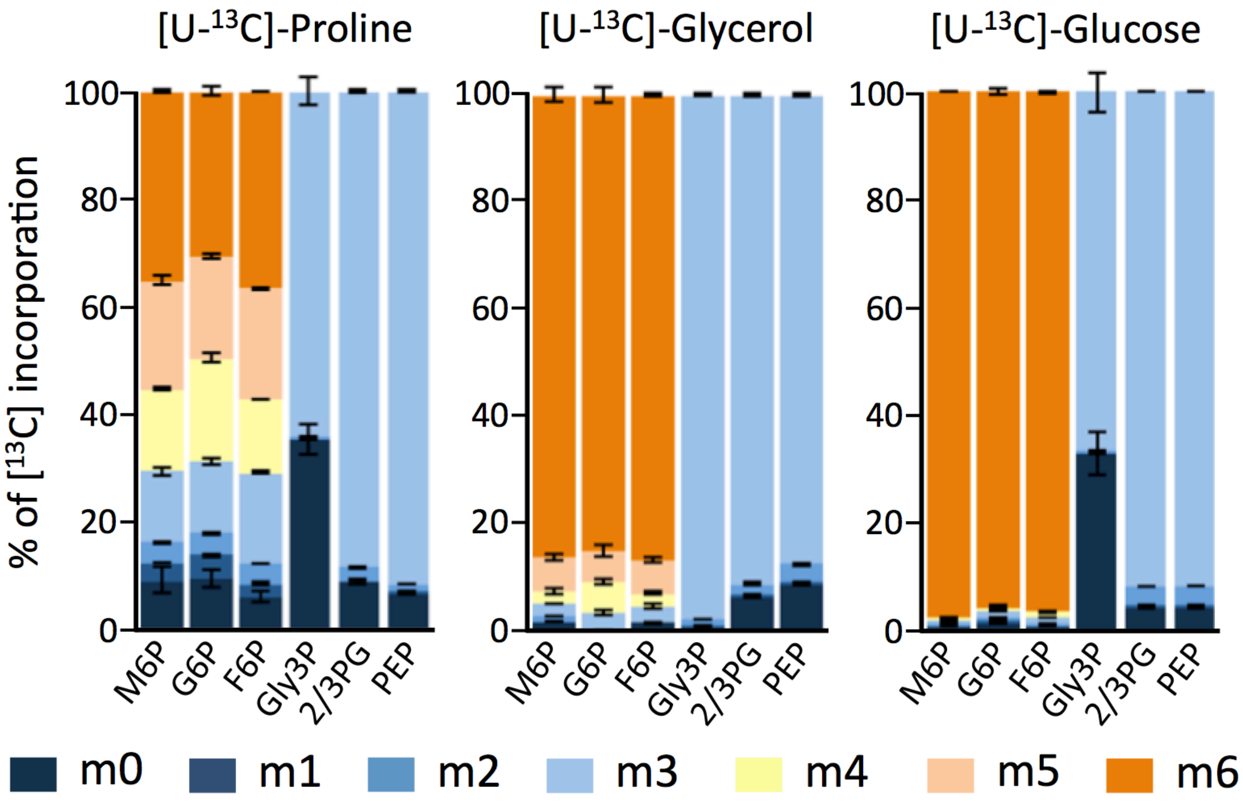

Supplement: S1 Fig — (PDF) [file ppat.1007502.s001.pdf]
