# Supplementary material for: Gluconeogenesis is essential for trypanosome development in the tsetse fly vector
Source: PLoS Pathog. 2018 Dec 17;14(12):e1007502. doi: 10.1371/journal.ppat.1007502 (PMC6312356; doi:10.1371/journal.ppat.1007502)

S2 Fig. The number of glycosomes is not affected by the *FBPase* gene knockout. The number of glycosomes in the parental (WT), Δ*fbpase* and Δ*fbpase*/FBPase cells grown in glucose-rich and glucose-free conditions was estimated by immunofluorescence using an immune serum against aldolase, a know glycosomal glycolytic enzyme. All cell lines were fixed with 2% formaldehyde in PBS, permeabilized with 0.2% Nonidet NP-40 and spread on poly-L-lysine coated slides. After incubation for 30 min in PBS containing 3% BSA, slides were incubated with rabbit anti-enolase (diluted 1:100) followed by ALEXA Fluor® 488-conjugated goat anti-rabbit secondary antibody (diluted 1:100) (Molecular Probes). Cells were viewed with a Leica DM5500B microscope and images were captured by an ORCA®-R2 camera (Hamamatsu) and Leica MM AF Imaging System software (MetaMorph®). Processing was performed with ImageJ.

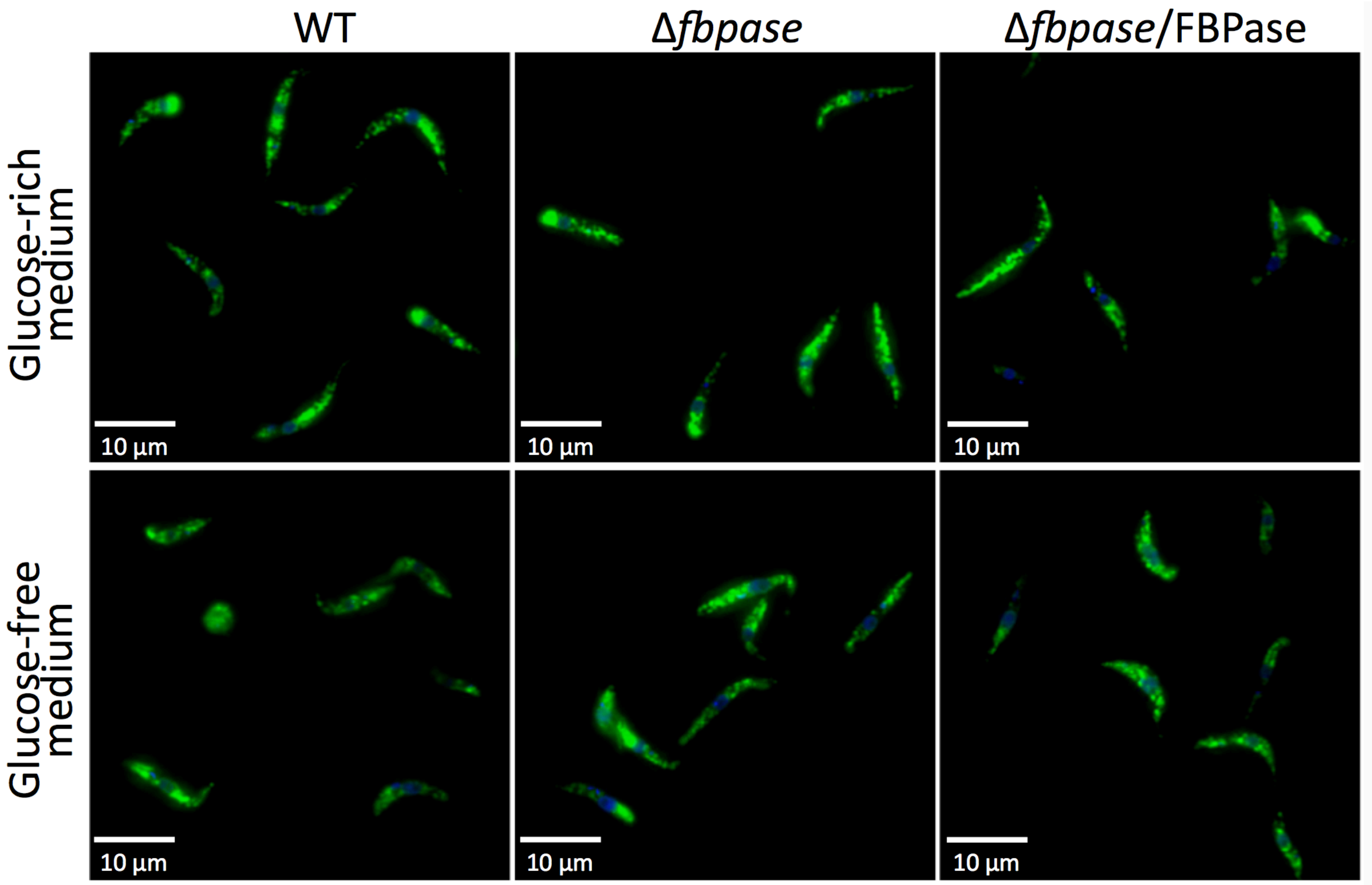

Supplement: S2 Fig — (PDF) [file ppat.1007502.s002.pdf]
